# Supplementary figures and images for: Loss of the abasic site sensor HMCES is synthetic lethal with the activity of the APOBEC3A cytosine deaminase in cancer cells
Source: PLoS Biol. 2021 Mar 31;19(3):e3001176. doi: 10.1371/journal.pbio.3001176 (PMC8041192; doi:10.1371/journal.pbio.3001176)

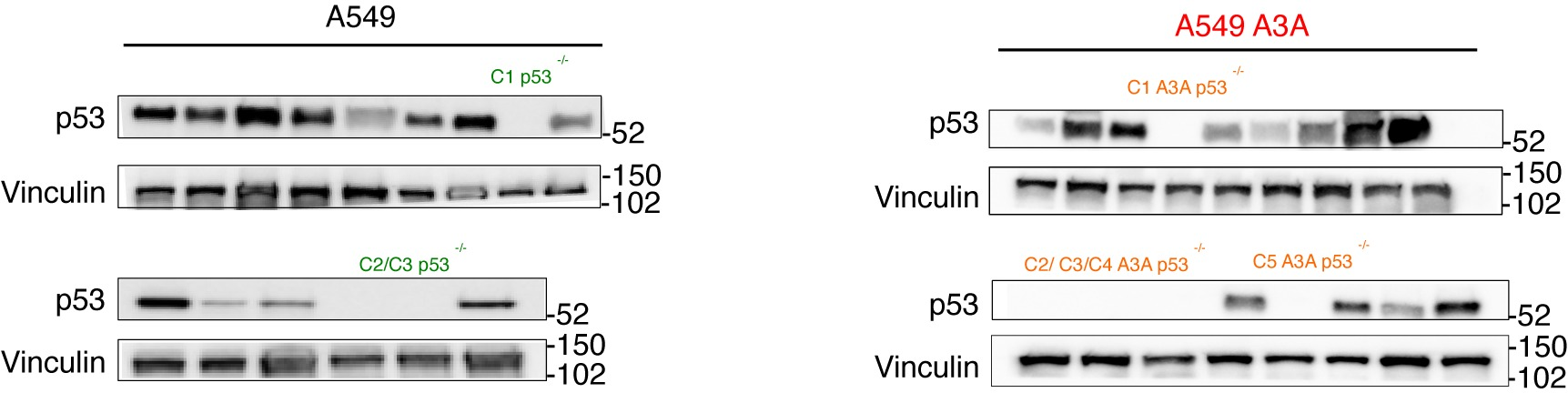

Supplement: S1 Fig — Western blot of the A549p53−/− (left panel) and the A549 A3A transduced p53−/− clones (right panel) generated using CRISPR/Cas-9 targeting. Uncropped blots are provided in S1 Raw Images. A3A, APOBEC3A. (TIF) [file pbio.3001176.s001.tif]

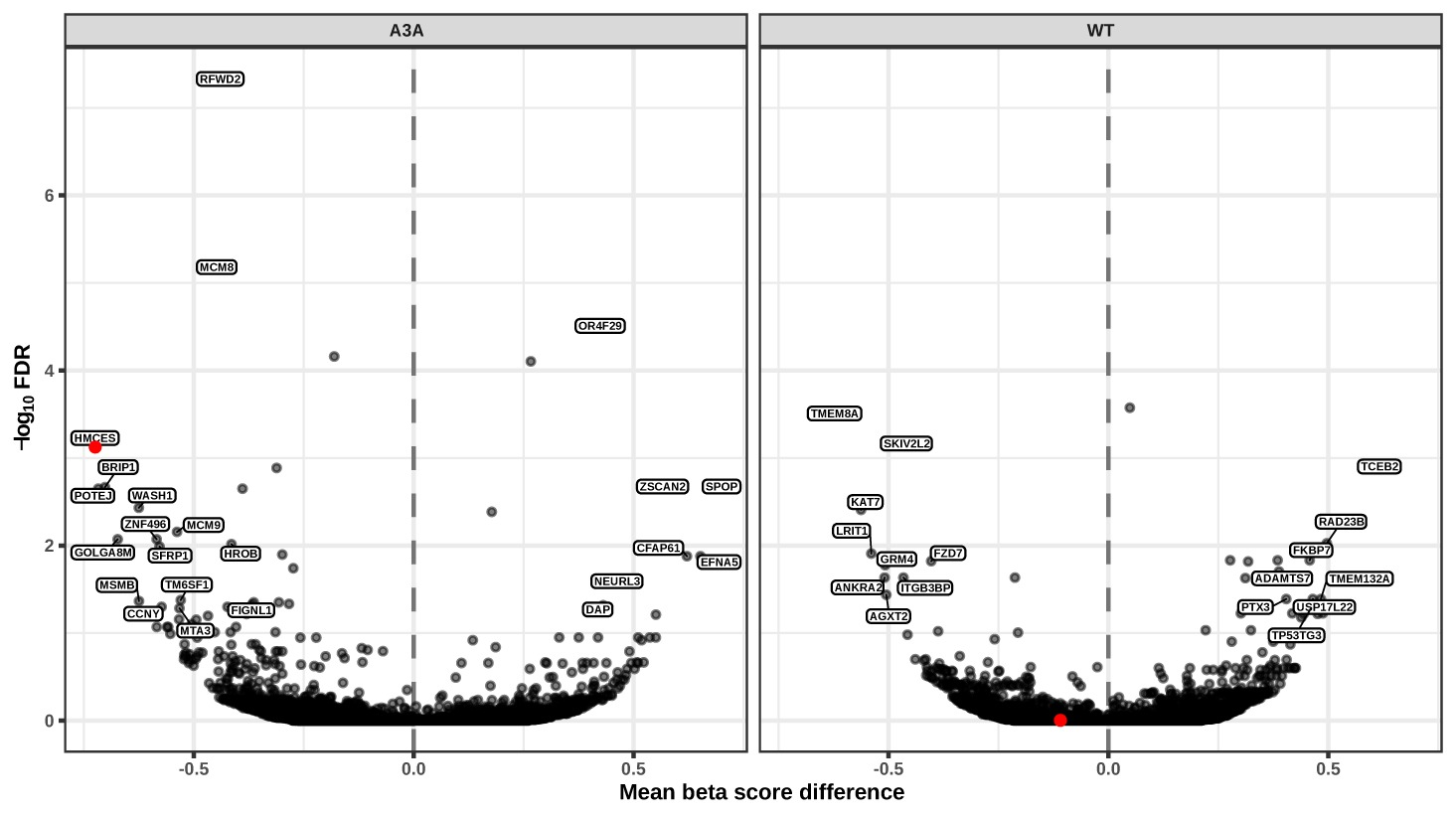

Supplement: S2 Fig — Volcano plot highlighting genes for which there could exist interaction with DOX per se. The x-axis represents the difference of the (normalized) MAGeCK-MLE’s beta score between treating a sample with DOX (IC25) and the corresponding control sample, averaged across the A3A plasmid-free version of all cell lines sampled after 15 days of cell culture. Intuitively, genes with significant negative beta score differences suggest conditional essentiality with DOX. The y-axis represents the −log10 FDR of the Fisher’s combined p-value (either lower or upper tail) across samples. Numerical data used for the plot are provided in S2 Data. A3A, APOBEC3A; DOX, doxycycline; FDR, false discovery rate. (TIF) [file pbio.3001176.s002.tif]

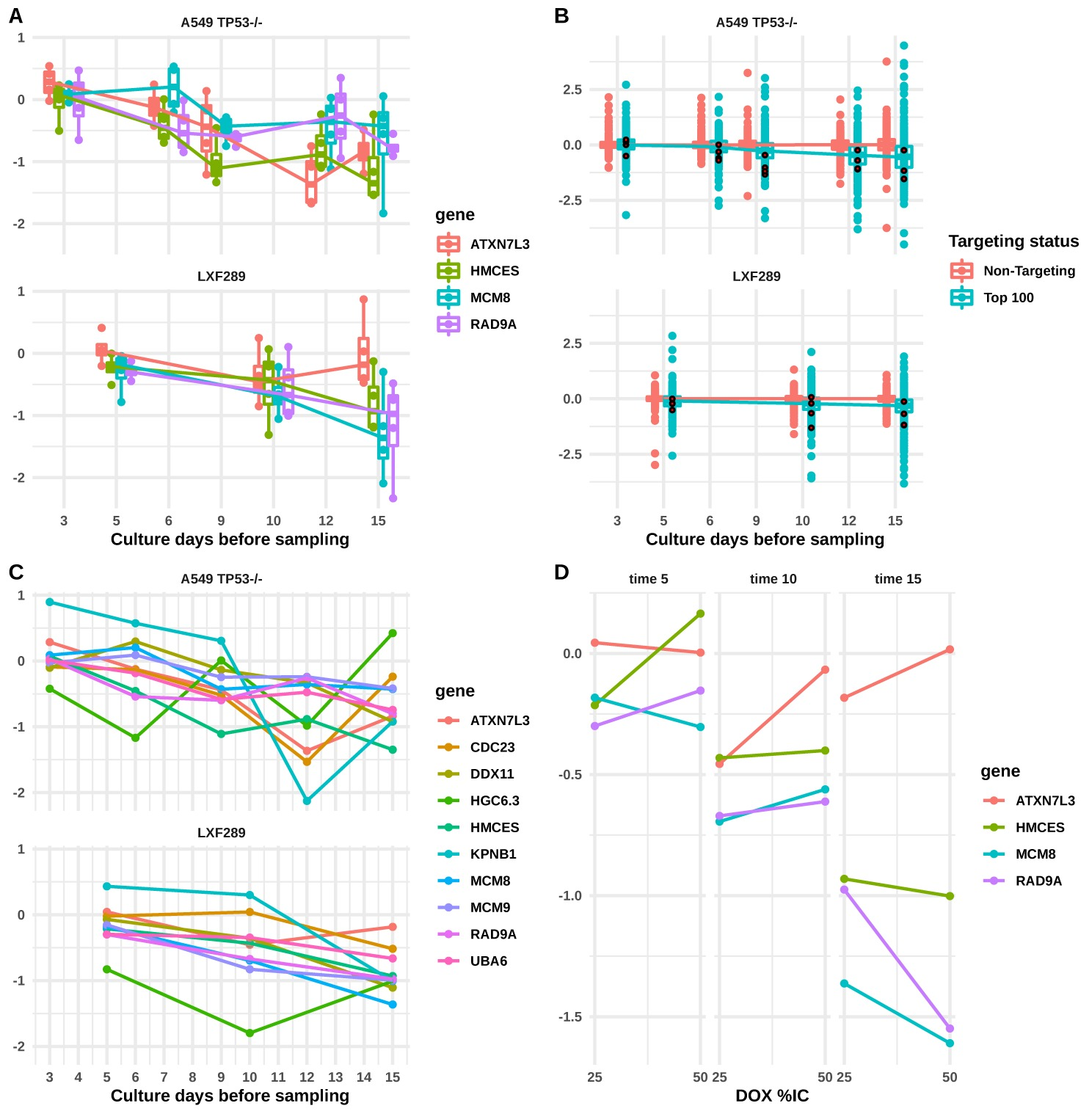

Supplement: S3 Fig — LFC (y-axes) represents the cell count differences between a sample treated with DOX (IC25 in plots A, B, and C) and the corresponding control (untreated) sample. (A) The top 4 genes are shown after sorting based on the overall score. The 4 sgRNAs targeting each gene are shown separately, and their count distribution is represented as a boxplot. Lines join the median sgRNA counts for each gene. One of the top 5 genes, HGC6.3, was excluded from the plot due to low data quality (see S1 Table). (B) The sgRNAs shown are the 1,000 nontargeting control sgRNAs in the Brunello library [50] and the top 100 genes after sorting genes based on the overall score. Lines join the median sgRNA counts for each distribution. Red dots indicate the LFC of sgRNAs for the HMCES gene. (C) The top 10 genes are shown after sorting by the overall score. Here, the HGC6.3 gene is included, while according to MAGeCK-MLE, it had low sgRNA efficiency (S1 Table), possibly causing the rather erratic trends across time points that it exhibits. (D) Difference in LFC dependent on DOX dose, either IC25 or IC50, in the LXF289 cell line. Columns show LFCs at different sampling times. The top 4 genes by overall score are shown. Numerical data used for all plots are provided in S3 Data. DOX, doxycycline; LFC, log2 fold change; sgRNA, single gRNA. (TIF) [file pbio.3001176.s003.tif]

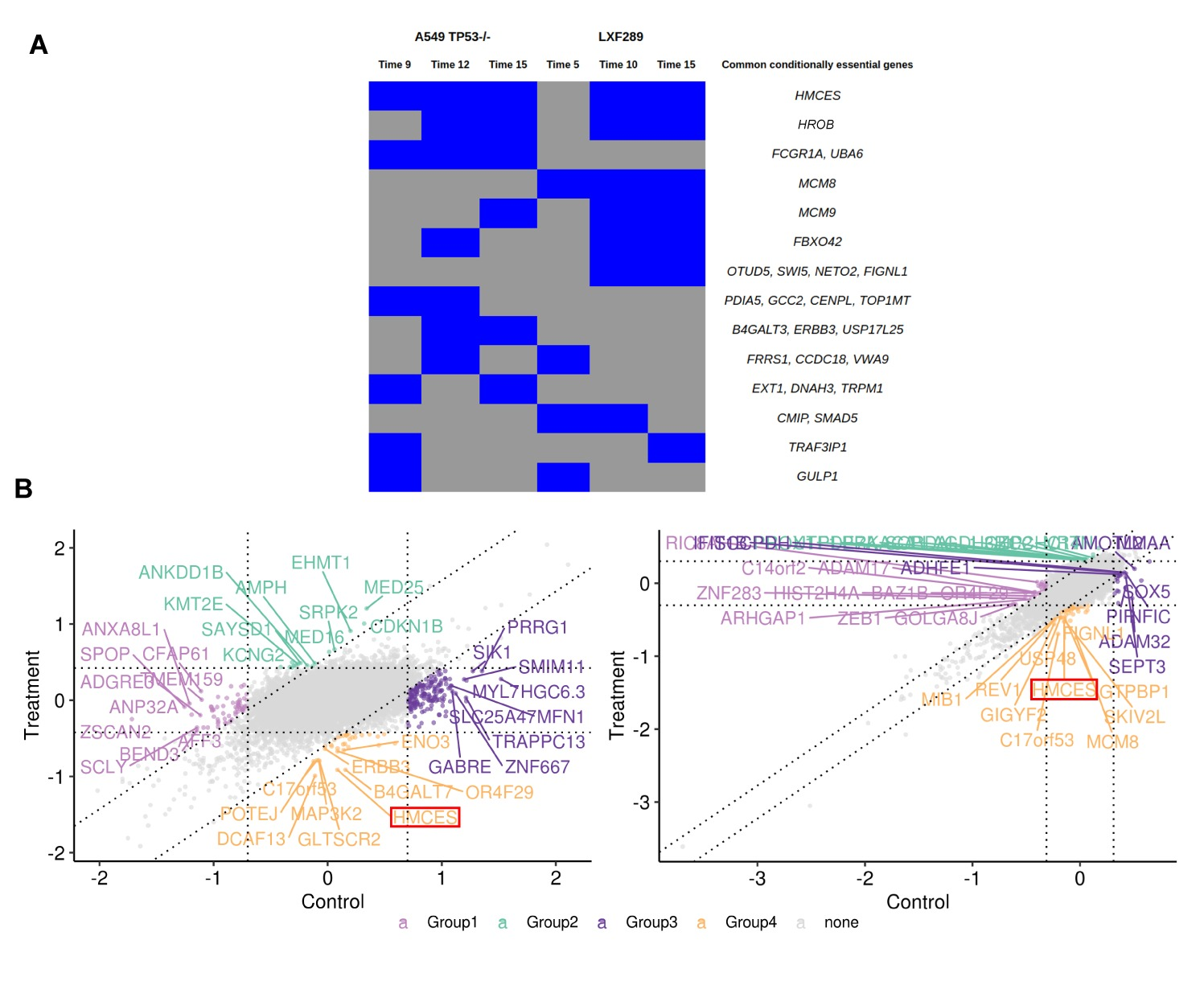

Supplement: S4 Fig — (A) Out of a total of 339 genes identified as conditionally essential by MAGeCK-MLE in either of the 2 cell lines examined (see next point), this figure shows those genes that were significant in more than 1 sample (time point/cell line combination). A blue box indicates that the genes in that row are conditionally essential (under A3A overexpression) in the corresponding sample, while a gray box indicates that there is no significant essentiality. HMCES is the gene found to be conditionally essential in the highest number samples—all samples except the earliest time point of LXF-289, time 5. (B) MAGeCK-MLE visualizations (“nine-square plots” as in [93]) based on (left) A549 TP53−/− and (right) LXF-289 cell lines, both sampled at day 15. Points represent genes distributed according to the between-samples normalized beta scores (enrichments) for the control sample (untreated, x-axis) and DOX-treated sample (at IC25 concentration, y-axis). Vertical and horizontal dotted lines indicate 2 SDs of the beta score distribution away from 0 to each side. Analogously, diagonal dotted lines represent 2 SDs of the distribution of between-treatment beta score differences away from 0 to each side. Therefore, genes located in the bottom center square (“Group4” genes) have MAGeCK beta scores different between the control and treated sample, being not different from 0 in the control (i.e., no evidence of selection) but significantly negative in the treatment (i.e., negatively selected); in other words, these genes are conditionally selected under A3A overexpression (DOX-induced). The top 10 genes are labeled in each square. HMCES is a top hit in both cell lines. Numerical data used for all plots are provided in S4 Data. A3A, APOBEC3A; DOX, doxycycline; SD, standard deviation. (TIF) [file pbio.3001176.s004.tif]

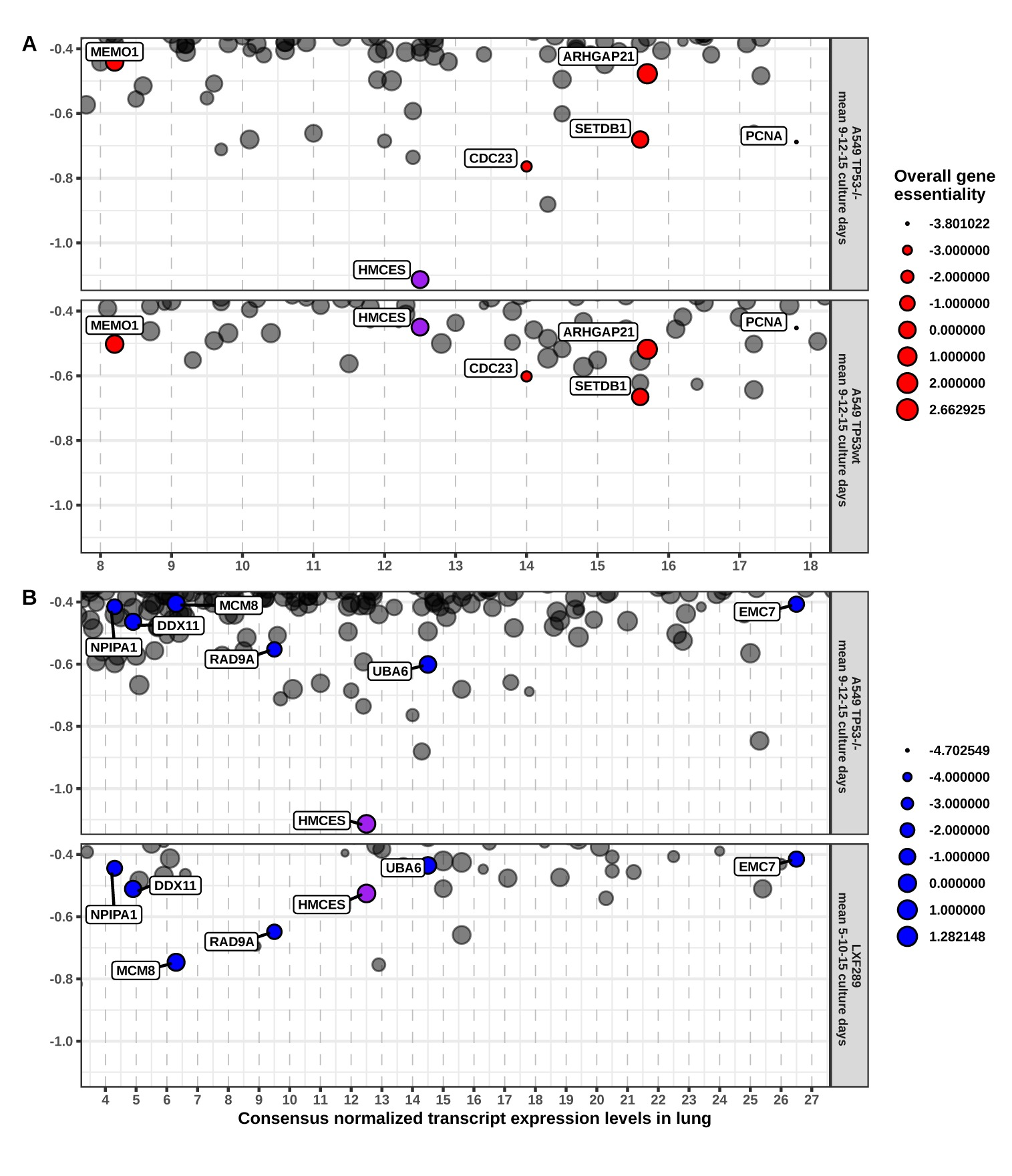

Supplement: S5 Fig — (A) Contrast between TP53 backgrounds of the A549 cell line. Red circles are genes with a consistently strong negative LFC (below −0.4) in both TP53 backgrounds (−/− above, wild-type below), considering the mean LFC after 9, 12, and 15 culture days (y-axes); LFC represents the cell count differences between a sample treated with DOX (IC25) and the corresponding control (untreated) sample. The circle area shows the beta score (enrichment) calculated with MAGeCK-MLE and averaged across the time points: A more negative beta score indicates stronger gene essentiality irrespective of treatment. X-axes represent the Human Protein Atlas consensus normalized (across cell lines) transcript expression levels (NX) in lung tissue for each gene. Among the hits, HMCES is prominent in the TP53−/− background but not in the wild-type background, has moderate expression levels in lung tissue, and does not appear to be generally strongly essential. (B) In an analogous manner, this plot shows the contrast of A3A conditionally essential genes between the A549TP53−/− and the LXF-289 genetic backgrounds; here, blue circles are genes with a consistently strong negative LFC (below -0.4) in both cell lines. Among the hits, HMCES, RAD9A and, to some extent, MCM8 appear consistent in both backgrounds; of these 3 genes, HMCES has somewhat higher expression levels in lung tissue and is the least essential in these cell lines. HMCES is the only hit that is consistent in both comparisons, and this is noted by using a purple color to highlight it. Numerical data for all plots are provided in S5 Data. A3A, APOBEC3A; DOX, doxycycline; LFC, log2 fold change. (TIF) [file pbio.3001176.s005.tif]

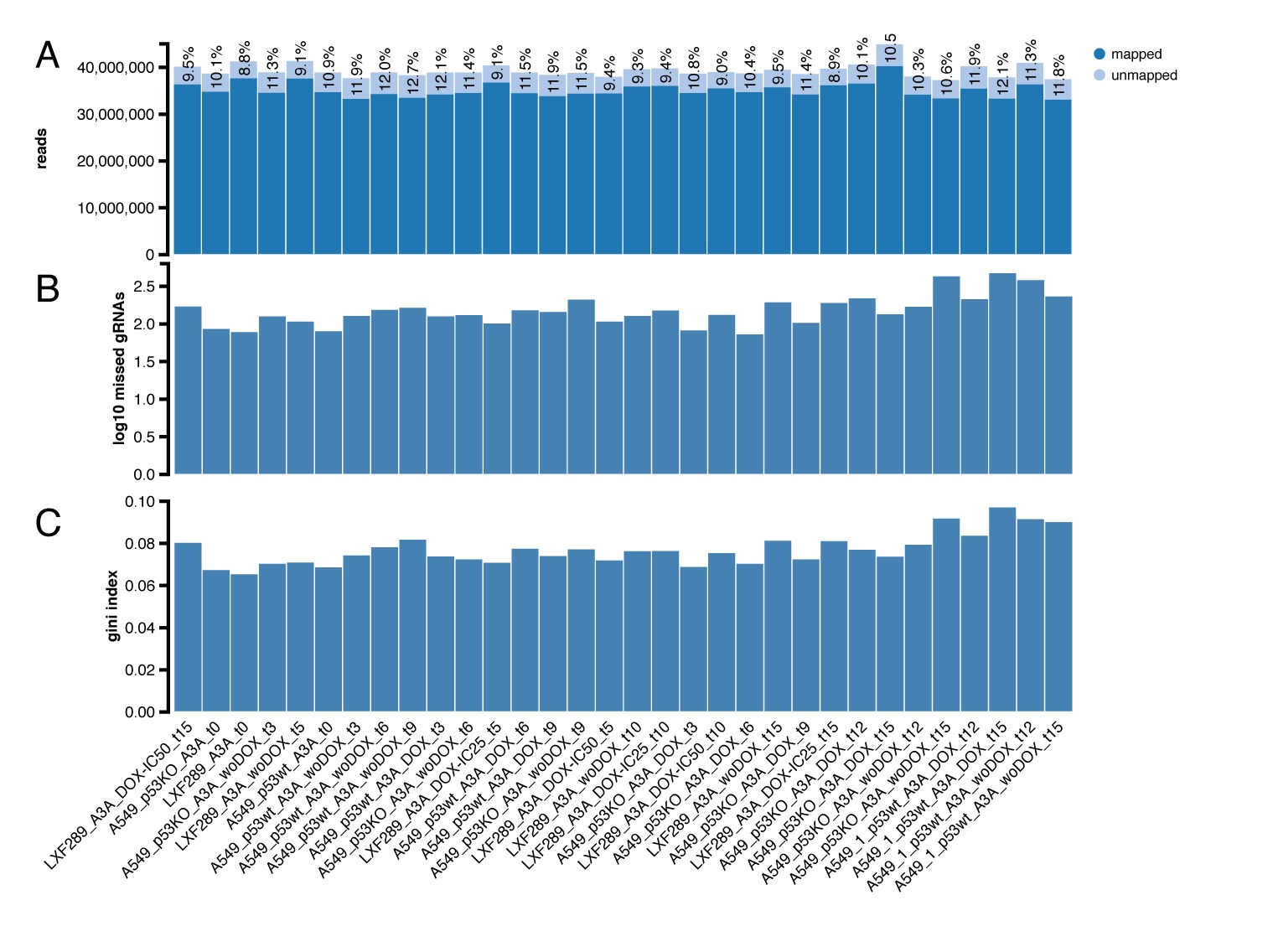

Supplement: S6 Fig — (A) Number of total sequenced reads per sample. Light blue fraction represents the percentage of reads that are unequivocally unmapped to the library, which is below the recommended maximum of 35% in all samples [51]. (B) Number of library sgRNAs that have 0 counts per sample. Figures are higher in late samples, but this is to be expected due to negative selection. Overall, sgRNAs with 0 counts are <1% of total sgRNAs in Brunello library (approximately 77K) [50,51]. Namely, the maximum number of sgRNAs is 461. (C) Gini index of log-scaled read count distributions. This measure of the evenness across all sgRNA counts is below the recommended maximum of 0.2 in all samples [51]. Also, the Gini index is expected to increase in later time points. Data used for plots are provided in S6 Data. QC, quality control; sgRNA, single gRNA. (TIF) [file pbio.3001176.s006.tif]

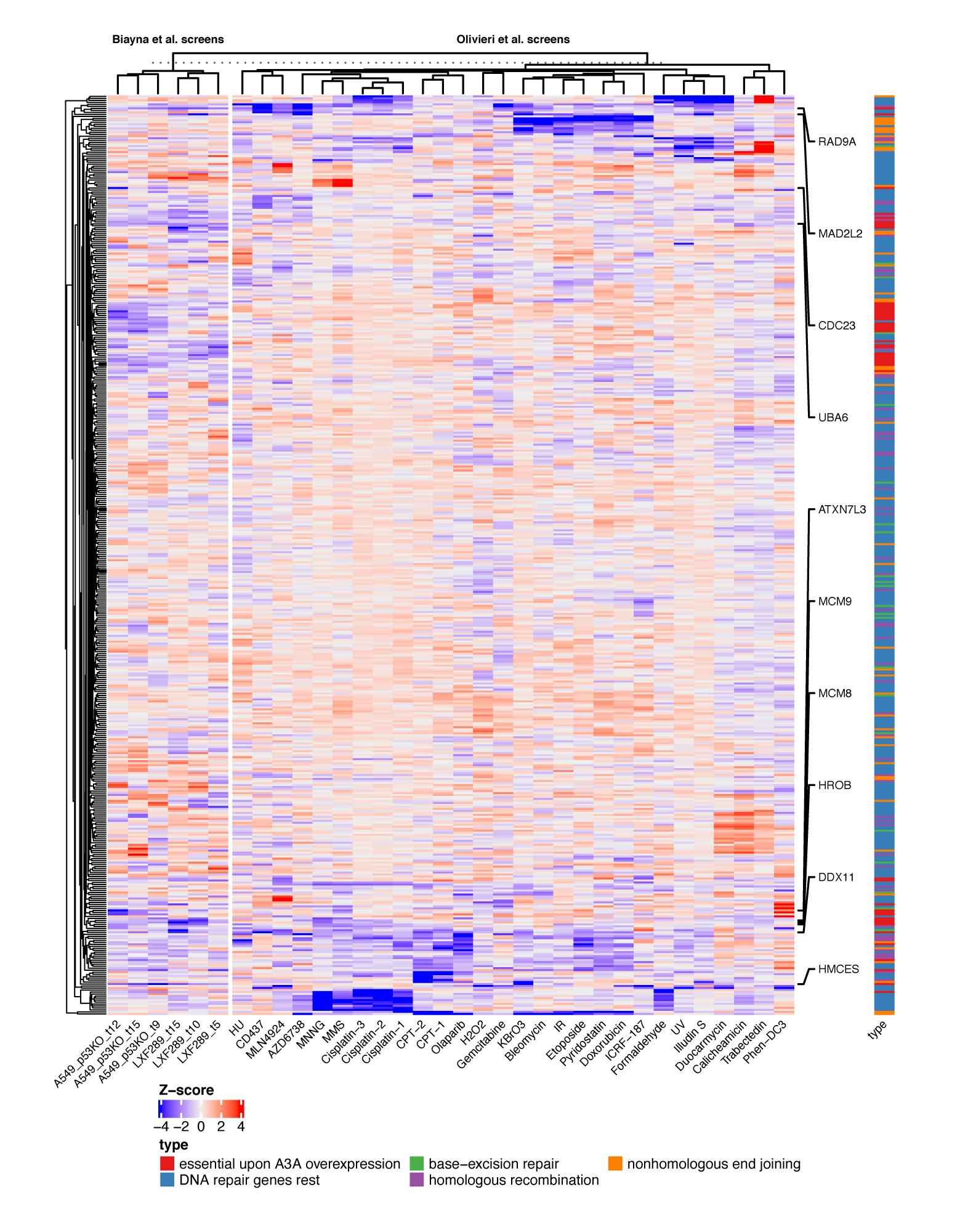

Supplement: S7 Fig — Differential fitness score (from project Achilles) upon APOBEC mutational signatures burden for the top 10 genes that are essential upon A3A overexpression in our screens (i.e., genes with the most negative mean LFC across 6 data points). Data used for the plots can be found in S5 Table. A3A, APOBEC3A; LFC, log2 fold change. (TIF) [file pbio.3001176.s007.tif]

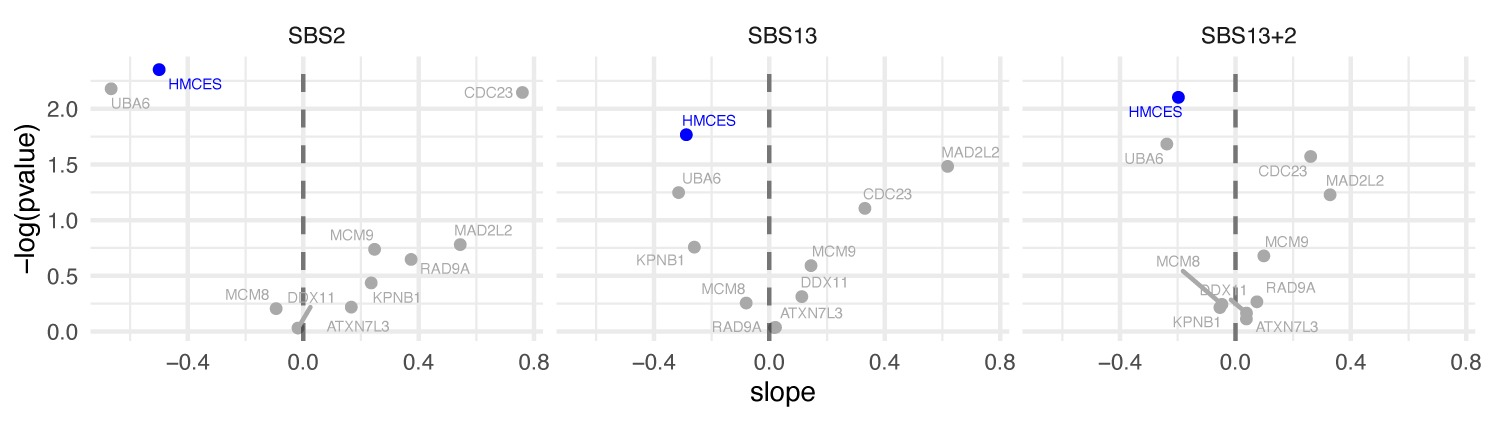

Supplement: S8 Fig — Left panel of heatmap shows a gene-level normalized LFC (gene essentiality score) upon A3A overexpression for 2 cell lines and for 3 time points (Biayna et al. screens); right panel shows Z-scores of gene essentiality after genotoxin exposure (Olivieri et al. screens) [60]. Data for 50 genes that are essential upon A3A overexpression in our screens (i.e., genes with the most negative mean LFC across 6 data points) (labeled “top”), and 521 DNA repair genes. Labels on the right-hand side highlight the 10 genes showing the highest overall A3A essentiality. Data used for the plots are provided in S7 Data. A3A, APOBEC3A; LFC, log2 fold change. (TIF) [file pbio.3001176.s008.tif]

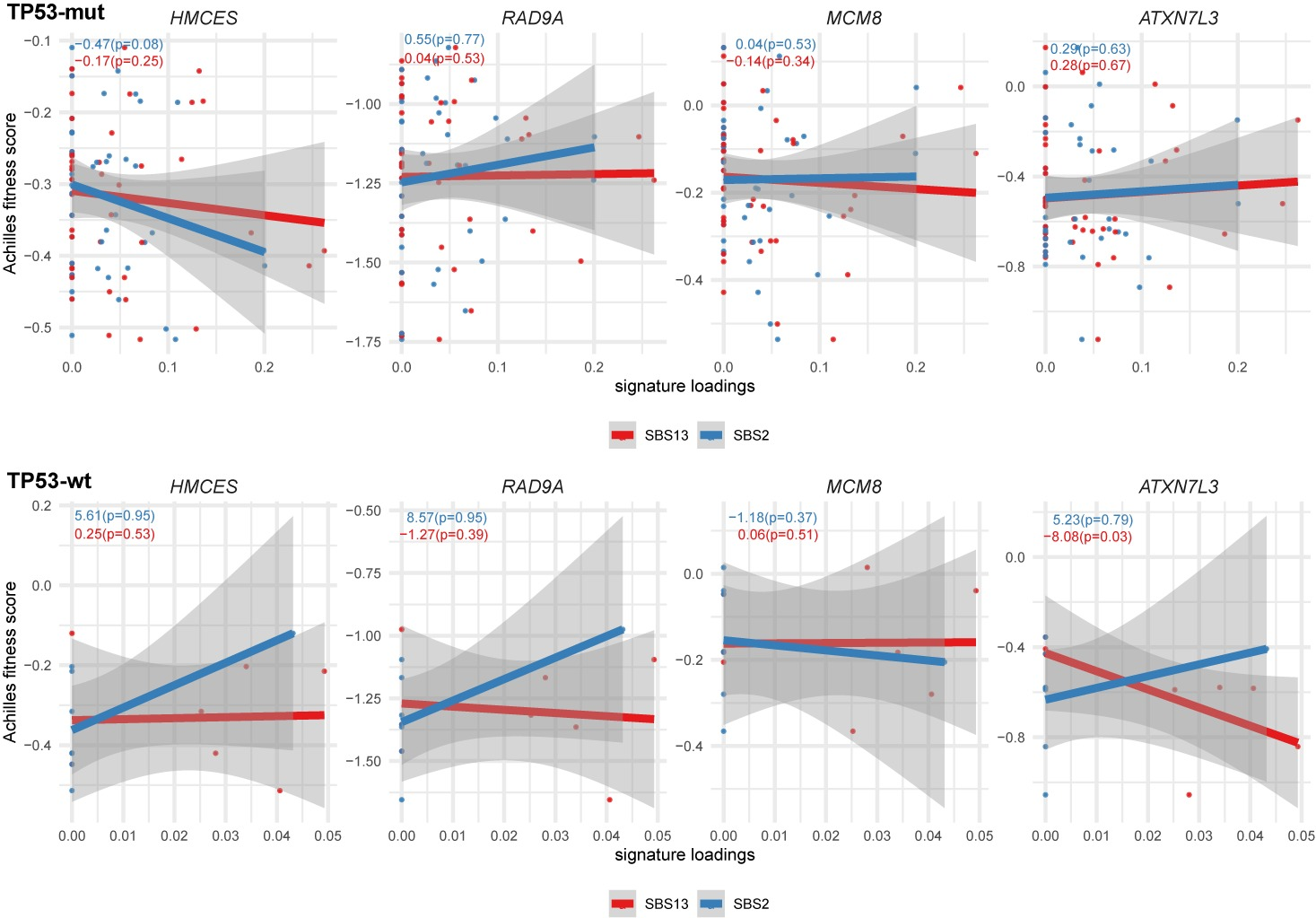

Supplement: S9 Fig — Cell lines originating from head and neck squamous cell carcinoma, LUAD, and lung squamous cell carcinoma were analyzed for the 4 genes with the greatest overall score in our genetic screens, while examining TP53 mutated (mut) and TP53 wt cell lines separately. The slope and p-value (1-tailed, lower) for the regression model for both APOBEC mutational signatures are shown within each panel. The more negative the slope, the more sensitive the cell lines are to the depletion of the particular gene at a higher level of the APOBEC mutational signature. Data used for the plots are provided in S7 Data. LUAD, lung adenocarcinoma; wt, wild-type. (TIF) [file pbio.3001176.s009.tif]

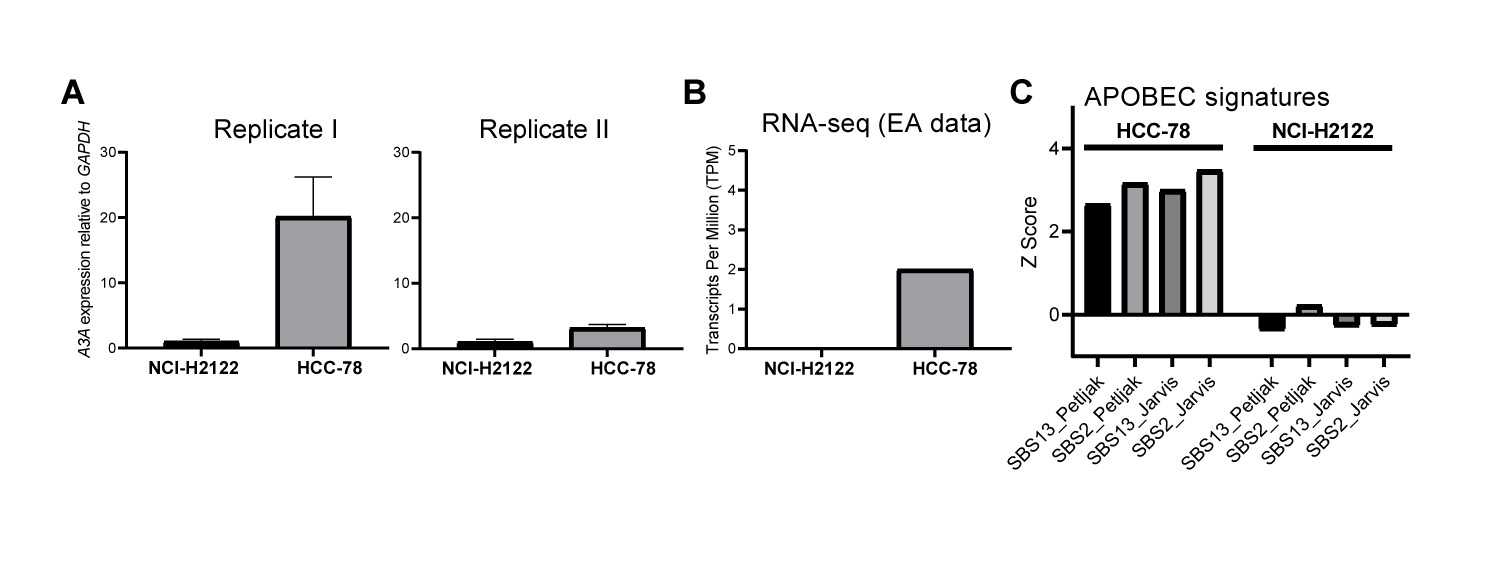

Supplement: S10 Fig — (A) Endogenous A3A mRNA expression levels in HCC-78 and NCI-H2122 cells relative to GAPDH measured by qRT-PCR (2 replicates). (B) A3A gene expression (TPMs) downloaded from EA (https://www.ebi.ac.uk/gxa/home) and (C) APOBEC mutational signatures (SBS2 and SBS13) burden downloaded from Petljak et al. and Jarvis et al. and normalized across cell lines (Z-score) [64,65]. Data used for the plots are provided in S7 Data. A3A, APOBEC3A; EA, expression atlas; qRT-PCR, quantitative real-time PCR. (TIF) [file pbio.3001176.s010.tif]

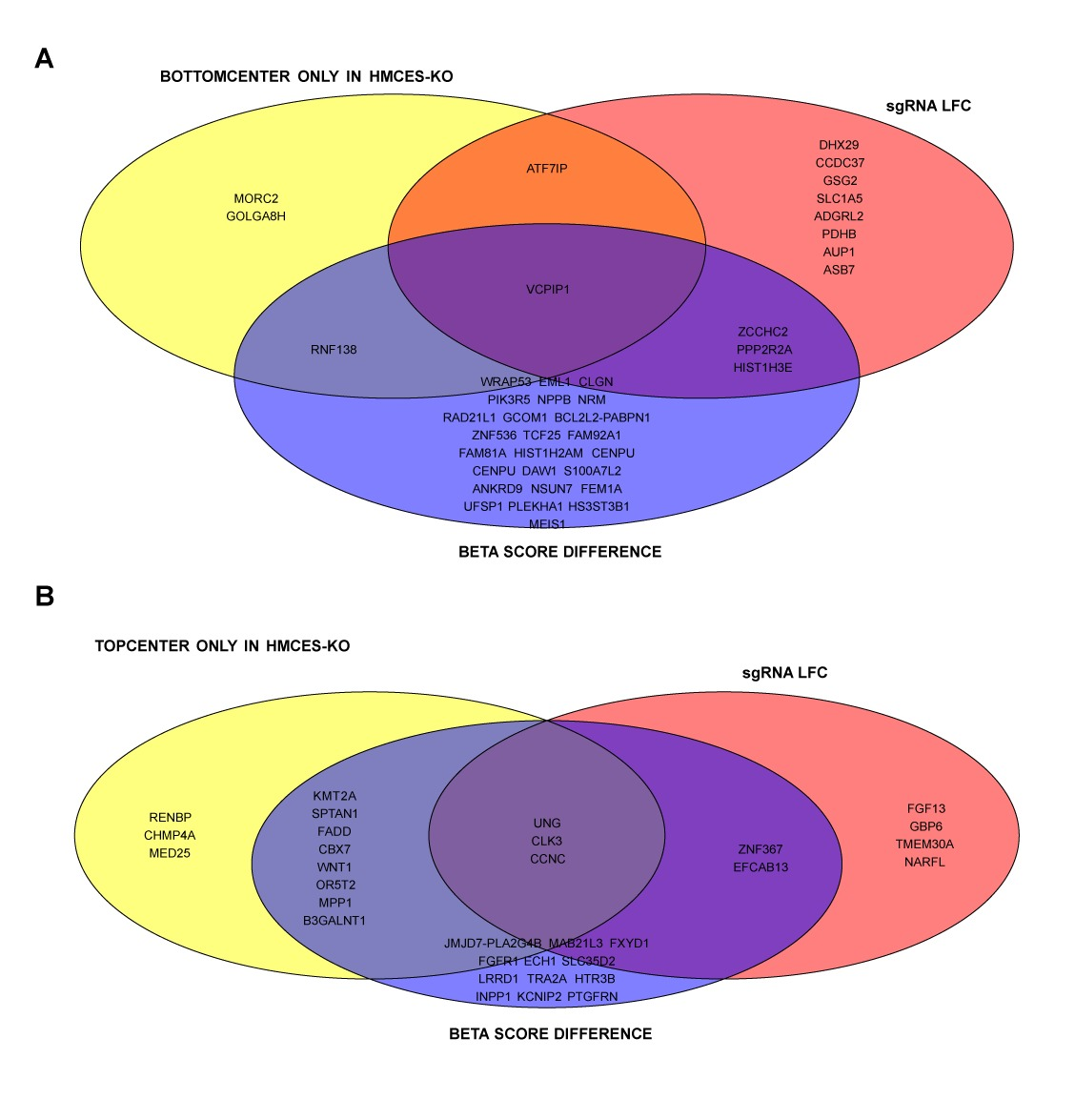

Supplement: S11 Fig — (A, B) Venn diagrams containing genes that are in epistasis with A3A expression (panel A, synthetic sickness/lethality, panel B, synthetic advantage) exclusively within an HMCES KO background, when applying 3 complementary statistical methodologies. Genes in the red circle have a standardized sgRNA LFC <-2 (A) or >2 (B) in the 4 DOX vs. control comparisons (IC25-t12, IC50-t12, IC25-t17, and IC50-t17) exclusively in HMCES KO samples. Genes in the blue circle fulfill the same criteria but using the MAGeCK-MLE standardized beta score difference, instead of the LFC. Lastly, the yellow circle contains genes whose normalized beta scores are not different from 0 in the control sample while they are significantly different from 0 (A, lower; B, higher) in the A3A-expressing sample, exclusively in an HMCES KO background: Specifically, this corresponds to the “bottom center” (A) or “top center” (B) square of MAGeCK-FLUTE’s nine-square scatterplot visualization (see panel B of S4 Fig). Data used for the plots are provided in S7 Data. A3A, APOBEC3A; DOX, doxycycline; KO, knockout; LFC, log2 fold change; sgRNA, single gRNA. (TIF) [file pbio.3001176.s011.tif]

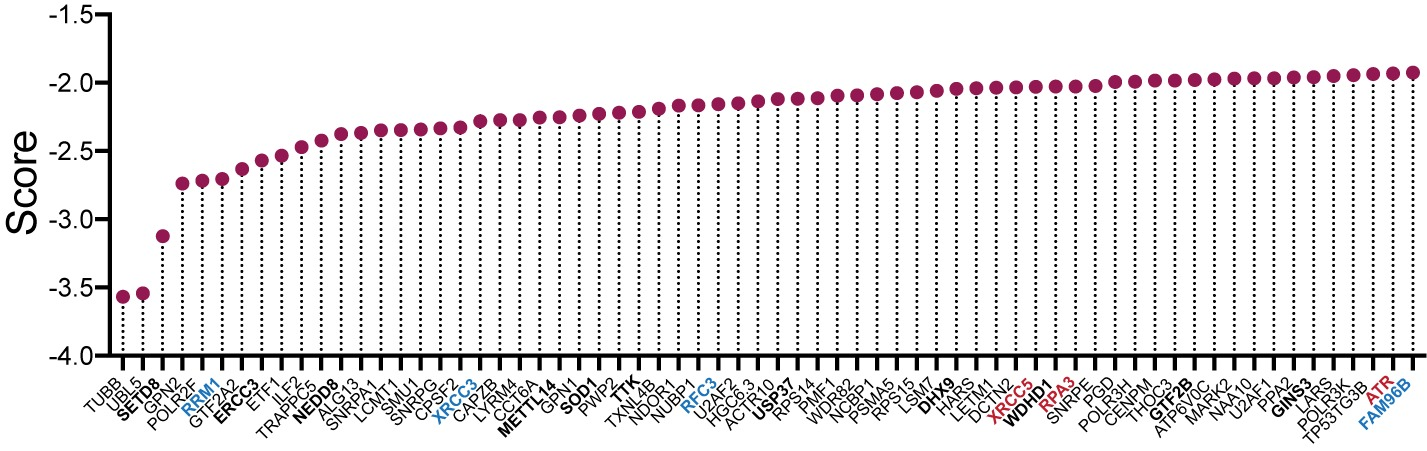

Supplement: S12 Fig — Plot depicts the overall score of the top 65 targeted genes that reduced the viability of LXF-289 HMCES KO cells using the mean LFC from 2 time point comparisons (LXF-289 A3A wt t10 vs. LXF-289 A3A HMCES KO t12 and LXF-289 A3A wt t15 vs. LXF-289 A3A HMCES KO t17). Genes implicated in the DNA damage response are in bold, PIKK substrates/regulators or kinases are in red, and those in common with a previously published screen in HEK-293 based HMCES KO cells (considering those genes with a negative p-value <0.05) are in blue [79]. Full data set used for the plots is provided in S7 Data. A3A, APOBEC3A; KO, knockout; LFC, log2 fold change; wt, wild-type. (TIF) [file pbio.3001176.s012.tif]
