# Supplementary material for: Loss of the abasic site sensor HMCES is synthetic lethal with the activity of the APOBEC3A cytosine deaminase in cancer cells
Source: PLoS Biol. 2021 Mar 31;19(3):e3001176. doi: 10.1371/journal.pbio.3001176 (PMC8041192; doi:10.1371/journal.pbio.3001176)
Supplement: S1 Table — Efficiencies of sgRNAs were estimated by the MAGeCK-MLE algorithm in A549TP53−/− and LXF-289 cell lines. Informally, the efficiencies estimate the probability that a given sgRNA is able to generate an inactivating double-strand DNA break in the targeted gene. Only the HGC6.3 gene shows overall low sgRNA efficiencies, particularly in the LXF-289 cell line, suggesting that the high log-fold change scores therein are an artefact (S3 Fig). HMCES has near-perfect sgRNA efficiencies. sgRNA, single gRNA. (PDF) [file pbio.3001176.s013.pdf]

| Gene | sgRNA | Cell line | Efficiency | | Gene | sgRNA | Cell line | Efficiency |
|---|---|---|---|---|---|---|---|---|
| | s_75152 | A549 TP53-/- | 1 | | | s_42031 | A549 TP53-/- | 1 |
| | | LXF289 | 0.727 | | | | LXF289 | 1 |
| | s_75153 | A549 TP53-/- | 1 | | | s_42032 | A549 TP53-/- | 1 |
| *HGC6.3* | | LXF289 | 0.596 | | *UBA6* | | LXF289 | 1 |
| | s_75154 | A549 TP53-/- | 0.977 | | | s_42033 | A549 TP53-/- | 1 |
| | | LXF289 | 0.602 | | | | LXF289 | 1 |
| | s_75155 | A549 TP53-/- | 1 | | | s_42034 | A549 TP53-/- | 1 |
| | | LXF289 | 0.644 | | | | LXF289 | 1 |
| | | | | | | | | |
| | s_44647 | A549 TP53-/- | 1 | | | s_4665 | A549 TP53-/- | 1 |
| | | LXF289 | 1 | | | | LXF289 | 1 |
| | s_44648 | A549 TP53-/- | 1 | | | s_4666 | A549 TP53-/- | 0.975 |
| *HMCES* | | LXF289 | 1 | | *DDX11* | | LXF289 | 1 |
| | s_44649 | A549 TP53-/- | 1 | | | s_4667 | A549 TP53-/- | 0.713 |
| | | LXF289 | 0.992 | | | | LXF289 | 0.999 |
| | s_44650 | A549 TP53-/- | 1 | | | s_4668 | A549 TP53-/- | 0.968 |
| | | LXF289 | 1 | | | | LXF289 | 1 |
| | | | | | | | | |
| | s_15717 | A549 TP53-/- | 1 | | | s_66465 | A549 TP53-/- | 0.996 |
| | | LXF289 | 1 | | | | LXF289 | 0.973 |
| | s_15718 | A549 TP53-/- | 1 | | | s_66466 | A549 TP53-/- | 0.998 |
| *RAD9A* | | LXF289 | 1 | | *MCM9* | | LXF289 | 1 |
| | s_15719 | A549 TP53-/- | 1 | | | s_66467 | A549 TP53-/- | 0.79 |
| | | LXF289 | 1 | | | | LXF289 | 1 |
| | s_15720 | A549 TP53-/- | 1 | | | s_66468 | A549 TP53-/- | 1 |
| | | LXF289 | 1 | | | | LXF289 | 1 |
| | | | | | | | | |
| | s_53711 | A549 TP53-/- | 1 | | | s_22709 | A549 TP53-/- | 1 |
| | | LXF289 | 1 | | | | LXF289 | 1 |
| | s_53712 | A549 TP53-/- | 0.956 | | | s_22710 | A549 TP53-/- | 1 |
| *MCM8* | | LXF289 | 0.999 | | *CDC23* | | LXF289 | 1 |
| | s_53713 | A549 TP53-/- | 0.937 | | | s_22711 | A549 TP53-/- | 1 |
| | | LXF289 | 1 | | | | LXF289 | 1.22E-13 |

| | | | | | | | | |
|---|---|---|---|---|---|---|---|---|
| | s_53714 | A549 TP53-/- | 0.976 | | | s_22712 | A549 TP53-/- | 1 |
| | | LXF289 | 1 | | | | LXF289 | 1 |
| | | | | | | | | |
| | s_44735 | A549 TP53-/- | 1 | | | s_28149 | A549 TP53-/- | 1 |
| | | LXF289 | 1 | | | | LXF289 | 1 |
| | s_44736 | A549 TP53-/- | 1 | | | s_28150 | A549 TP53-/- | 1 |
| *ATXN7L3* | | LXF289 | 1 | | *MAD2L2* | | LXF289 | 1 |
| | s_44737 | A549 TP53-/- | 1 | | | s_28151 | A549 TP53-/- | 1 |
| | | LXF289 | 1 | | | | LXF289 | 1 |
| | s_44738 | A549 TP53-/- | 1 | | | s_28152 | A549 TP53-/- | 1 |
| | | LXF289 | 1 | | | | LXF289 | 1 |
| | | | | | | | | |
| | s_10269 | A549 TP53-/- | 1 | | | | | |
| | | LXF289 | 1 | | | | | |
| | s_10270 | A549 TP53-/- | 1 | | | | | |
| *KPNB1* | | LXF289 | 1 | | | | | |
| | s_10271 | A549 TP53-/- | 1 | | | | | |
| | | LXF289 | 1 | | | | | |
| | s_10272 | A549 TP53-/- | 1 | | | | | |
| | | LXF289 | 1 | | | | | |
