# Supplementary material for: Loss of the abasic site sensor HMCES is synthetic lethal with the activity of the APOBEC3A cytosine deaminase in cancer cells
Source: PLoS Biol. 2021 Mar 31;19(3):e3001176. doi: 10.1371/journal.pbio.3001176 (PMC8041192; doi:10.1371/journal.pbio.3001176)
Supplement: S4 Table — AUC per sample, based on the capacity of the CRISPR screening to discriminate between known sets of essential [93–95] and nonessential [94] genes by their normalized read counts. For comparison, the AUCs for the same overall sets of genes in the genetic screens (RPE1 cell line) from Brown et al. (2019) [54] have been included: Note that, while our screening was based on the Brunello library[50], Brown et al. employed the TKO library, so the gene overlap is not total. AUC, Area under the receiving operating characteristic curves. (PDF) [file pbio.3001176.s016.pdf]

**Cell line**	**TP53**	**treatment**	**t9**	**t12**	**t15**	
	**KO**	**Control**	0.799	0.831	0.835	
		**DOX-IC25**	0.793	0.810	0.829	
**A549 A3A**						
	**wt**	**Control**	0.831	0.847	0.859	
		**DOX-IC25**	0.727	0.832	0.837	
			**t5**	**t10**	**t15**	
		**Control**	0.792	0.857	0.887	
**LXF289 A3A**	**mut**	**DOX-IC25**	0.789	0.854	0.889	
		**DOX-IC50**	0.779	0.847	0.885	
			**t9**	**t12**	**t15**	**t18**
	**KO**					0.881
**RPE1**[52]						
	**wt**		0.850	0.877	0.881	0.895
