# Supplementary material for: Loss of the abasic site sensor HMCES is synthetic lethal with the activity of the APOBEC3A cytosine deaminase in cancer cells
Source: PLoS Biol. 2021 Mar 31;19(3):e3001176. doi: 10.1371/journal.pbio.3001176 (PMC8041192; doi:10.1371/journal.pbio.3001176)
Supplement: S5 Table — Differential fitness scores (from project Achilles) upon APOBEC mutational signatures (SBS2, SBS13, and SBS13+2) burden for the top 10 genes that are essential upon A3A overexpression in our screens (i.e., genes with the most negative mean LFC across 6 data points). A3A, APOBEC3A; LFC, log2 fold change. (PDF) [file pbio.3001176.s017.pdf]

gene	signature	Regression coefficient	p-value (two-tailed)	pvalue (one-tailed, lower i.e. signature sensitizes to gene k.o.)
**HMCES**	SBS2	-0.499	0.095	0.048
UBA6	SBS2	-0.666	0.113	0.057
**HMCES**	SBS13+2	-0.197	0.122	0.061
**HMCES**	SBS13	-0.288	0.171	0.085
UBA6	SBS13+2	-0.238	0.186	0.093
UBA6	SBS13	-0.314	0.287	0.144
KPNB1	SBS13	-0.260	0.469	0.234
MCM8	SBS13	-0.080	0.774	0.387
MCM8	SBS13+2	-0.047	0.783	0.392
KPNB1	SBS13+2	-0.054	0.806	0.403
MCM8	SBS2	-0.094	0.814	0.407
DDX11	SBS2	-0.018	0.969	0.485
RAD9A	SBS13	0.018	0.965	0.517
ATXN7L3	SBS13	0.021	0.963	0.518
ATXN7L3	SBS13+2	0.038	0.893	0.553
DDX11	SBS13+2	0.038	0.847	0.576
ATXN7L3	SBS2	0.166	0.803	0.598
RAD9A	SBS13+2	0.075	0.766	0.617
DDX11	SBS13	0.113	0.731	0.634
KPNB1	SBS2	0.236	0.646	0.677
MCM9	SBS13	0.144	0.554	0.723
RAD9A	SBS2	0.374	0.524	0.738
MCM9	SBS13+2	0.099	0.507	0.746
MCM9	SBS2	0.247	0.478	0.761
MAD2L2	SBS2	0.544	0.458	0.771
CDC23	SBS13	0.331	0.331	0.834
MAD2L2	SBS13+2	0.328	0.293	0.854
MAD2L2	SBS13	0.618	0.227	0.886
CDC23	SBS13+2	0.261	0.208	0.896
CDC23	SBS2	0.760	0.117	0.942
