# Supplementary material for: Loss of the abasic site sensor HMCES is synthetic lethal with the activity of the APOBEC3A cytosine deaminase in cancer cells
Source: PLoS Biol. 2021 Mar 31;19(3):e3001176. doi: 10.1371/journal.pbio.3001176 (PMC8041192; doi:10.1371/journal.pbio.3001176)
Supplement: S8 Table — List of qRT-PCR primers (TaqMan/oligonucleotides) and PCR primers for library amplification and NGS. Primer sequence obtained from PrimerBank denoted with an * [96]. NGS, next-generation sequencing; qRT-PCR, quantitative real-time PCR. (PDF) [file pbio.3001176.s020.pdf]

**Taqman Probes**	
**Gen Name**	Probe ID
HMCES	Hs99999905_m1
GAPDH	Hs99999905_m1
**Oligonucleotides**	
**Gene Name**	Sequence (5’-3’) / Sets
APOBEC3A Forward	TGGCATTGGAAGGCATAAGAC*
APOBEC3A Reverse	TTAGCCTGGTTGTGTAGAAAGC*
GAPDH Forward	AGCCACATCGCTCAGACAC
GAPDH Reverse	GCCCAATACGACCAAATCC
PCR1 Forward	AATGATACGGCGACCACCGAGATCTCGATTTCTTGGCTTTATATATCTTGTGGAAAGGACG
PCR1 Reverse	GTGACTGGAGTTCAGACGTGTGCTCTTCCGATCTCCAATTCCCACTCCTTTCAAGACCT
Illumina Forward (PCR2)	AATGATACGGCGACCACCGAGATCT
NEBNext Multiplex Oligos (Reverse) (PCR2)	E7335S (Set 1), E7500S (Set 2), E7710S (Set 3) i E7730S (Set 4)
Custom Sequencing Primer	CGATTTCTTGGCTTTATATATCTTGTGGAAAGGACGAAACACCG
