# Supplementary material for: Loss of the abasic site sensor HMCES is synthetic lethal with the activity of the APOBEC3A cytosine deaminase in cancer cells
Source: PLoS Biol. 2021 Mar 31;19(3):e3001176. doi: 10.1371/journal.pbio.3001176 (PMC8041192; doi:10.1371/journal.pbio.3001176)

Fig 1B uncropped blots

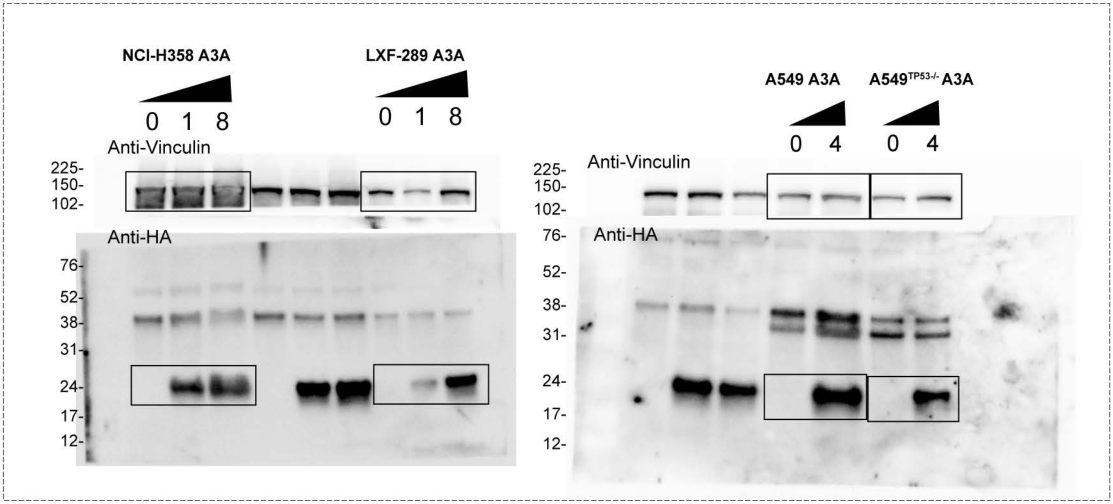

Fig 4A uncropped blots

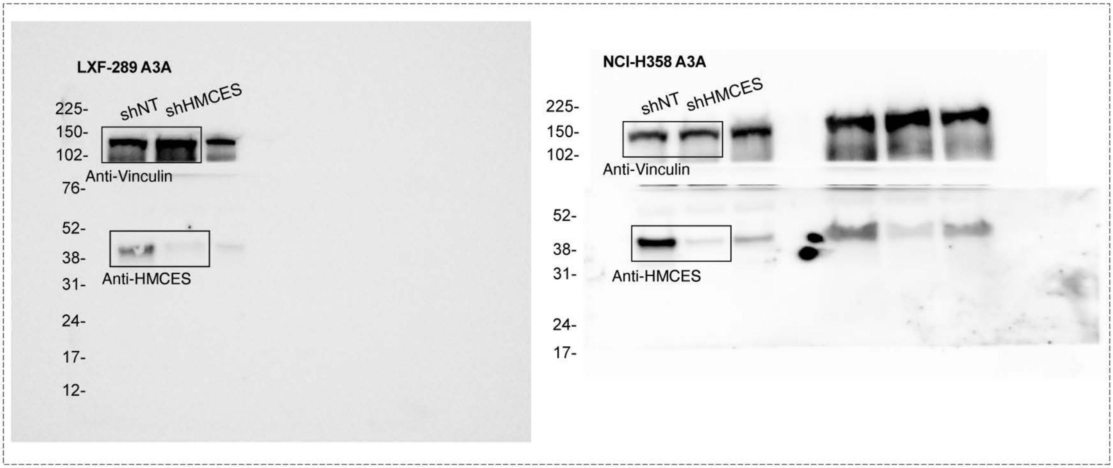

Fig 4D uncropped blots

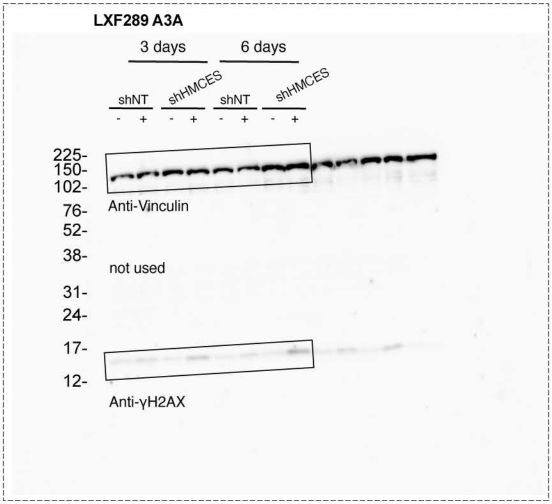

Fig 4I uncropped blots

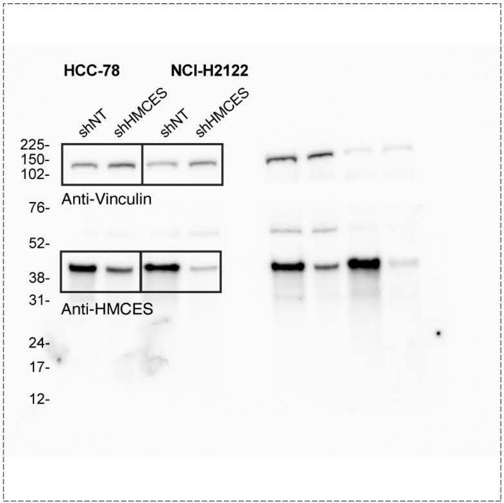

Fig 5B uncropped blots

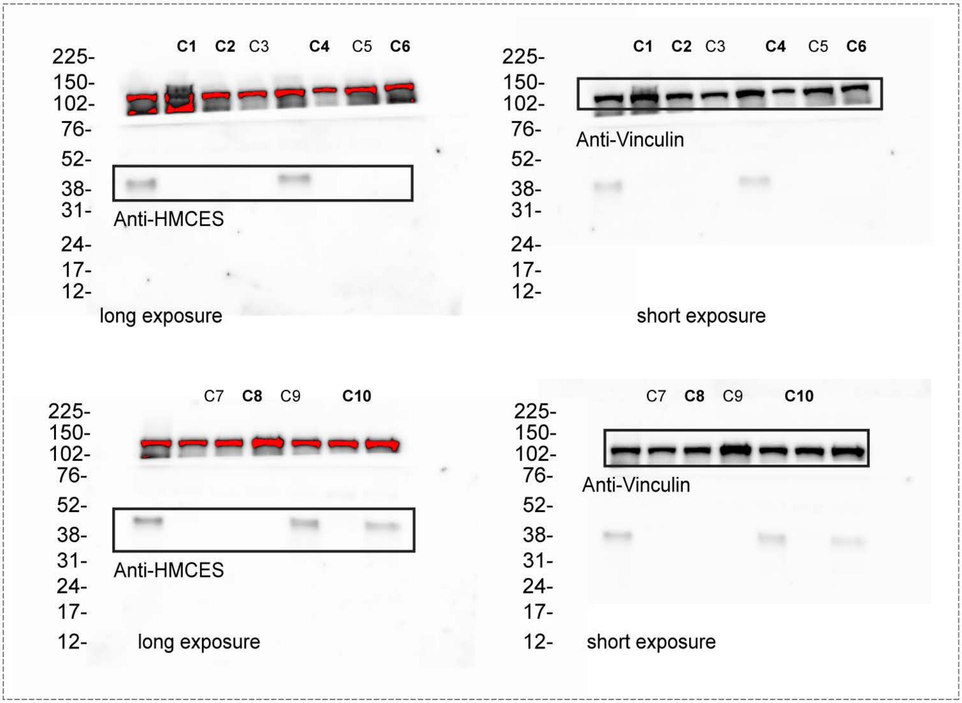

S1 Fig uncropped blots

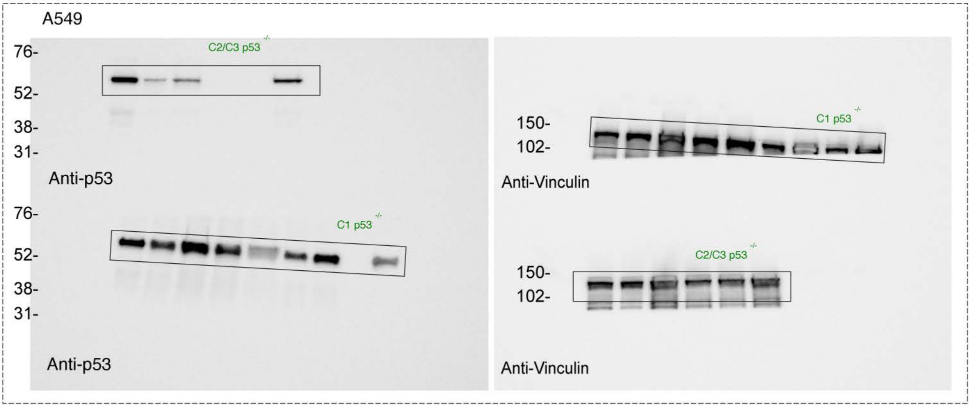

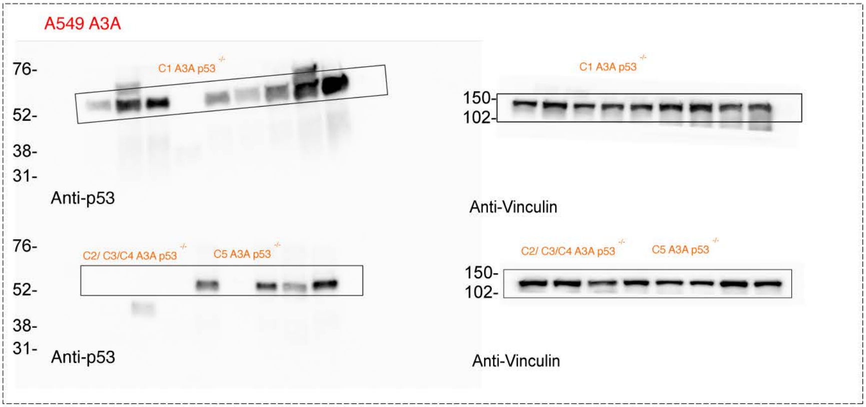

Supplement: S1 Raw Images — (PDF) [file pbio.3001176.s033.pdf]
