# Supplementary material for: Loss of the abasic site sensor HMCES is synthetic lethal with the activity of the APOBEC3A cytosine deaminase in cancer cells
Source: PLoS Biol. 2021 Mar 31;19(3):e3001176. doi: 10.1371/journal.pbio.3001176 (PMC8041192; doi:10.1371/journal.pbio.3001176)
Supplement: Attachment — Submitted filename: Reviewer Repsonses A3A__0117.docx [file pbio.3001176.s034.docx]

**Response to reviewers (PBIOLOGY-D-20-02244R1):**

We would like to thank the reviewers for their supportive and constructive comments on our manuscript. We included a point-by-point response to all comments below, as well as some new data to support our responses, including a secondary screen in HMCES KO cells that we believe adds additional mechanistic insight and intriguing hits for future analysis. To accommodate these data and requested changes, we extensively edited the manuscript and figures and provided a copy with all changes denoted in red and the revised figures in the text to aid the reviewer’s assessment. These documents are also submitted separately conforming to the journal formatting requirements. We hope that the reviewers will now find the revised manuscript acceptable for publication in PLOS Biology.

Our point-by-point response (in blue) to reviewer comments (italicized) follows.

**Reviewer #1:**

*Upregulation of APOBEC3A and APOBEC3B contribute to mutagenesis in certain types of cancer. Thus, finding vulnerabilities of tumours with high expression of A3A/A3B may open the door to novel targeted therapies. APOBEC3 activity induces abasic sites by cytosine deamination, leading to high levels of mutations and replicative stress. However, the genes and pathways involved in the repair of DNA damage induced by A3A/A3B overexpression are not yet known. Recent works have shown that HMCES is a DNA repair factor which binds covalently to abasic sites induced by exogenous oxidative damage, promoting microhomology-mediated end joining and avoiding mutagenic pathways during replication.*

*In this work, Byana and colleagues employed an unbiased functional genomic approach to find genetic vulnerabilities using two A549 and LXF-289 lung adenocarcinoma cell lines conditionally expressing APOBEC3A, putting forward a set of target proteins. The screening approach is well executed, obtaining a dataset enriched in DNA repair genes, as expected. They focused on HMCES, providing experimental evidence that cells lacking functional levels of HMCES are hypersensitive to A3A overexpression, accumulate DNA damage with defects in cell cycle progression. They finally proposed that HMCES is an attractive target for p53-mutated/APOBEC3A overexpressing tumors, demonstrating that the efficacy of this approach synergizes in combination with other previously proposed therapeutic approaches such as ATRi inhibition.*

*The main problem with this manuscript is the fact that the vulnerability of APOBEC3A-overexpressing cells to the loss of HMCES was recently reported and mechanistically defined to greater depth by Mehta et al, 2020 (PMC7313144). While it will be useful to the field that two groups independently arrived at the conclusion that A3A-expressing cells require HMCES for optimal fitness, it is difficult to be enthusiastic about this manuscript in the absence of additional insights, mechanistic or translational.*

While some of the novelty of our findings linking HMCES to A3A mediated damage was lost with the publication of the paper from the Cortez lab(Mehta et al. 2020), we feel that our paper indeed adds a number of additional insights that will be of interest to the field and justify its publication. In addition to replicating the exciting conclusion that HMCES promotes tolerance of A3A-mediated damage, we report the first screen for A3A sensitivity in multiple cell lines, including an TP53 wild-type and TP53 KO isogenic pair, and extensive computational analysis of mutational signatures in cell line genomes that further extends these results to 76 cell lines analyzed in Project Achilles. In addition to HMCES, we report numerous additional candidate targets for modulating sensitivity to A3A expression, as well as new data from secondary screens in HMCES KO cells that we have added in revision, revealing many genetic interactions of HMCES with other genes, specifically in the context of A3A activity. In particular, this screen identified UNG as positively selected- indicating its activity drives the sensitivity of HMCES KO cells to A3A-activity. Also notable was the identification of TDP1, that can facilitate repair of AP-sites, and VCPIP1, that modulates the activity of multiple enzymes that repair DNA-protein crosslinks, as sensitizers of HMCES KO cells expressing A3A. This indicates that TDP1 is likely a key backup pathway and further implicates AP-sites, that are a target if its activity, in the toxicity of A3A expression in HMCES KOs. We have included additional description of this screen in the results and discussion but include it here for the reviewers:

**Reviewer Fig 1 (now Fig 6 in revised manuscript): Secondary screening identifies modifiers of the response to A3A in HMCES KO LXF-289** **cells.** (**A**) Schematic of the secondary screen in HMCES KO LXF-289 cells. (**B**) Principal component (PC) analysis of A3A-conditional LFC scores for all genes across all 10 experimental conditions (see labels next to arrows, which show loadings of the conditions on PC1 and PC2; HMCES wt refers to parental LXF-289 cell line. Top genes, prioritized by score are highlighted on the figure for each indicated comparison: green, alleviating epistasis; black, aggravating epistasis. Gene level scores and GO analysis are included in S6 Table and S7 Table and additional epistasis analysis in S11 Fig in the revised manuscript.

We agree with the reviewer that the overlap between our work and that of the Cortez lab is beneficial for the field and we have further highlighted our findings in light of those of the Cortez lab in the revised discussion, as appropriately suggested by one of the reviewers. We feel that the publication of our work will improve scientific reproducibility and hope that we can share our robust genetic screening dataset soon through publication in PLOS Biology.

*Other specific comments:*

*1)	To further support a role for targeting HMCES in A3A-overexpressing cells, it would be interesting to assess whether the catalytic activity of HMCES is involved in promoting the fitness of A3A-expressing cells.*

While the enzymatic activity of HMCES was shown to be important in the context of some types of damage other than that generated by A3A (Mohni et al. 2019), it has been proposed to be dispensable for repair during CSR (Shukla et al. 2020). Thus, we agree this is an interesting experiment to perform in the context of A3A.

We attempted this experiment using HMCES expression constructs that worked well in 293 cells but we could not detect expression in transduced LXF-289 cells that we have used for the majority of the revision experiments. We presume that this is a promoter issue and are trying a number of things to troubleshoot this, but it could take a significant amount of additional time (2-3 months), as we have to remake and test lentivirus constructs with different promoters in multiple cell lines, or make HMCES KOs in additional cell lines where we can express the cDNA (293s etc). Given that neither of our labs can operate at full capacity, it is hard to be sure how soon we can complete this.

While we will continue to pursue this experiment, as well as further analysis of pathways identified in the secondary screening, we feel that the enzymatic activity is very likely to be required based on the available evidence and the result would not alter our conclusions overall, regardless of the outcome. We would therefore prefer not to further delay review and publication of the manuscript for this result, as we know another manuscript reporting APOBEC3 screening is currently being evaluated. We will try to incorporate this in the final version if we can complete the experiments in time and if the reviewers are favorable about how other points have been addressed in the revised manuscript.

2)	Similarly, the authors should demonstrate experimentally that silencing A3A in a cancer cells displaying a APOBEC mutation signature reduces dependency on HMCES.

This is a logical experiment but is not trivial to perform. A3A expression has been reported to be episodic (Petljak et al. 2019), due to unknown factors, and current reagents for detecting endogenous A3A protein levels are poor. However, we have several lines of evidence indicating that the sensitivity of HMCES KO cells is dependent on A3A expression.

1. In our revised manuscript, we report a secondary screen in HMCES KO cells. A3A is among the top hits in this screen, indicating that its expression drives cell death in the HMCES KO background (Fig 6 of revised mansuscript).
2. We reported an analysis of 76 cell lines from the DepMap project and showed that loss of fitness associated with HMCES deletion is higher in cells with A3A mutational signatures (“SBS2” and “SBS13”) (Fig 3A). We acknowledge that this is indirect evidence, but nevertheless represents robust experimental evidence supporting the proposition that cells with the A3 mutational signature are more dependent on HMCES.
3. Lastly, we have performed several additional experiments to address this point explicitly. We depleted HMCES in 2 cell lines with low (H2122) or high (HCC78) endogenous levels of A3A (estimated by real time PCR and previous work) and analyzed sensitivity to shRNA depletion of HMCES. We found that cells with higher A3A levels were more sensitive to HMCES depletion (revised Fig 4G-I and S10 Fig- combined in the reviewer figure below). In addition, we titrated down the DOX levels in our LXF-289 A3A cells to achieve A3A induction levels comparable to the endogenous levels of NCI-H358 cells. Using this condition, we again see significantly increased sensitivity to A3A induction specifically in the HMCES depletion condition. Together these experiments indicate that our experimental results are reproducible in a range of expression that is comparable to the endogenous levels of some cell lines and that cells lacking HMCES are more sensitive to endogenous levels of A3A expression.

**Reviewer Fig 2 (panels are now in Fig 4 and S10 Fig of the revised manuscript.** (**A**) Endogenous *A3A* mRNA expression levels in HCC-78 and NCI-H2122 relative to *GAPDH* measured by quantitative real-time PCR (two independent biological replicates). (**B**) A3A gene expression (TPMs) downloaded from expression atlas (EA; https://www.ebi.ac.uk/gxa/home) and (**C**) APOBEC mutational signatures (SBS2 and SBS13) burden downloaded from Petljak et al. and Jarvis et. al and normalized across cell lines (z-score)(Petljak et al. 2019; Jarvis et al. 2018). (**D**) Growth inhibition (percentage of growth rate) for the indicated cell lines transduced with shNT or shHMCES. HMCES levels are shown, Vinculin is used as a loading control. (**E**) *A3A* mRNA expression levels in LXF-289 (0 and 0.125 ug/ml of doxycycline) and the parental NCI-H358 cell line relative to *GAPDH* measured by quantitative real-time PCR. qPCR analysis were repeated two times, mean and SEM are shown. (**F**) Depletion of HMCES sensitizes LXF-289 A3A cells to A3A expression following low levels of DOX treatment. Statistical analysis of shHMCES versus shNT in all panels was performed using a one-tailed unpaired *t*-test; error bars indicate SD. *, p ≤ 0.05. For each time point, growth assays and clonogenic survival assays in D and F were repeated 3 times.

*3)	The authors state that 'HMCES depletion results in synthetic lethality with A3A expression specifically in a TP53-mutant background'. This claim relies solely in the interpretation of their screening data in A549 cells and lacks experimental validation. As this one of the few novel aspects of this manuscript, it should be validated experimentally.*

This claim relies not only on the interpretation of our screening data, but also on the analysis of the DepMap data where we have information about the effects of HMCES depletion on survival, as well as the A3 mutational signature status and p53 status. To further highlight this point, we have included an additional analysis of this data plotting the influence of HMCES loss depending on A3-signature status in p53 mutant or wt cell lines (revised S9 Fig). We include this figure here for the reviewer. It shows that the negative slope of the regression line – meaning: higher sensitivity to HMCES KO in Project Achilles cell lines with higher levels of A3 mutational signatures – is seen only in TP53 mutant cell lines but not TP53 wild-type cell lines (we do acknowledge that the number of TP53 wt cell lines in our panel is rather small however it is clear that they do not support the general trend seen in the TP53 mutants).

**Reviewer Fig 3 (now S9 Fig in revised manuscript). Gene essentiality fitness score from project Achilles versus APOBEC mutational signatures exposures.** Cell lines originating from head-and-neck squamous cell carcinoma, lung adenocarcinoma, and lung squamous cell carcinoma were analyzed for the four genes with the greatest overall score in our genetic screens, while examining TP53 mutated (mut) and TP53 wild-type (wt) cell lines separately. The slope and p-value (one-tailed, lower) for the regression model for both APOBEC mutational signatures are shown within each panel. The more negative the slope, the more sensitive the cell lines are to the depletion of the particular gene at a higher level of the APOBEC mutational signature.

In addition to this data analysis, we performed additional experiments. In particular we validated this conclusion using matched p53 WT and KO A549 cell lines and clonogenic survival assays (see revised Fig 4F and individual data panel below). While induction of A3A expression reduced viability of A549 cells, there is no significant effect of HMCES depletion on viability. In contrast, HMCES depletion in A549-p53 KO cells causes a strong reduction in viability following A3A expression. In summary, we feel this conclusion is well supported by our screening, the 76 analyzed genetic screens from the DepMap database, and direct experimental evidence in matched cell lines presented here.

**Reviewer Fig 4 (now Fig 4F in the revised manuscript).** The effect of HMCES depletion is TP53-dependent. Clonogenic survival assays of A549 or A549^TP53-/-^ cells are shown 10 days after treatment with the indicated dose of DOX. Statistical analysis of shHMCES versus shNT in all panels was performed using a one-tailed unpaired *t*-test; error bars indicate SD. *, p ≤ 0.05, **, p ≤ 0.01, ***, p≤ 0.001. For each time point, growth assays and clonogenic survival assays were repeated 3 times.

*4)	In a similar vein, what is the impact of the p53 status on the analysis of dependencies across the 76 lung and head-and-neck cancer cell lines with respect to HMCES essentiality?*

This was addressed in the response to the previous comment and associated S9 Fig.

*5)	We strongly encourage the authors to provide, at minimum, the gene-level depletion scores for their screens so they can be independently analyzed.*

We agree this is important and have provided several supplemental tables with: (i) raw, sgRNA level data (counts of sequencing reads), and additionally (ii) gene level data from MAGECK analysis, showing the log-fold change (“lfc”) scores that were used to derive the PCA plots in our manuscript. We provide this data for the initial and secondary screens in the revised submission (S2 Table, S6 Table and S9 Table in revised manuscript).

*6)	The use of time-dependent depletion is an interesting approach to select for true-positives. However, I wonder if it improves the detection of "core" essential genes, which could be used to validate this approach. One worry I have is that time-dependency may favor the identification of sgRNAs that target genes that code for unstable proteins or messenger mRNAs as those may be the first to show depletion at the earliest time point.*

We understand the reviewer is concerned about the mRNA/protein turnover rates confounding our analysis of gene essentiality, which uses multiple time points.

This is a good point however it does not apply to our experimental design, where we measure only the *conditional* gene essentiality (comparing A3A-expressing cells versus non-expressing cells at the same time), rather than the absolute gene essentiality. To show this, , we have included an analysis of the time-dependent depletion (the “log fold change” [LFC] statistic) of core essential(Hart et al. 2017) and non-essential genes(Hart et al. 2014), as well as the 1000 non-targeting sgRNAs in the Brunello library, shown both for the LXF-289 and the A549 cell lines (Reviewer Fig 5). It is clear from the plots that the LFC of all of these sets of genes hardly changes during the course experiment, as expected because the A3A condition is always normalized to the control. This is in contrast to the HMCES and the other top-4 genes that become strongly depleted with time. We hope this answers the reviewer’s concern. We are happy to do further analyses if the reviewer deems necessary.

**Reviewer Fig 5.** The conditional essentiality upon A3A overexpression (measured as log_2_ fold change between treatment and control conditions at the same time point) of *HMCES, ATXN7L3, RAD9A* and *MCM8* genes, compared with sets of essential genes, non-essential genes, and non-targeting sgRNAs.

*Minor issues*

*-	Fig. 2B lacks a label on the X-axis*

We have corrected the axis label, thanks for pointing this out.

*-	On p4, the authors write MCM8/9-HROB/MCM8IP: this is confusing as it seems to suggest that HROB and MCM8IP are different proteins*

We agree and have revised the nomenclature to use HROB consistently and we have added the alternative gene names for HROB at its first use to avoid confusion.

**Reviewer #2:**

*Biayna and coworkers perform a very interesting set of CRISPR screens to identify genetic dependencies with overexpression of the single-stranded DNA deaminase APOBEC3A (A3A). A strength of the study is screening in multiple lines as well as in the presence/absence of p53, which has been shown previously to influence CRISPR results as well as A3A induced genotoxicity. Results with artificial overexpression are very clear. However, a major weakness limiting overall impact is not showing that cancer cell lines with endogenous A3A levels have evolved to be dependent on HMCES function. As it stands, artificially high A3A levels may cause genotoxic stresses that are not normally occurring/accumulating in tumor cells.*

1)	This manuscript shows that A3A overexpression (by Dox system) leads to a synthetic dependency requiring HMCES expression. This genetic interaction may be artifactual and a critical missing experiment is demonstrating that endogenous A3A levels (or "naturally" upregulated levels in cancer lines) are also synthetic with endogenous HMCES expression. In other words, is the viability of cancer cell lines with endogenous A3A expression similarly dependent upon HMCES?

As we previously mentioned, the lack of sensitive reagents to track endogenous A3A protein and the poor understanding of how the episodic expression of A3A(Petljak et al. 2019) is regulated make it challenging to address whether cancer cells with high endogenous A3A levels have evolved dependence on HMCES. Despite these limitations, we have tried to address this very interesting point in several ways.

As we presented in Reviewer Fig 2, we performed new experiments to examine the sensitivity of an endogenous A3A-expressing NSCLC line (HCC-78) to HMCES depletion, compared to a non-expressing cell line (NCI-H2122) and found that the A3A expressing line was sensitive. We also performed new experiments to examine the endogenous A3A mRNA levels in NCI-H358 expressing cell lines using real-time PCR and titrated down our dox induction to maintain *A3A* expression levels in a physiological range in LXF-289 cells and still observed sensitivity to A3A induction in HMCES depleted cells. We also point out that this these experiments are well supported by our previous observational analysis of the DepMap data, where the effect of HMCES loss on fitness was measured across 76 cell lines (see Reviewer Figure 3 and S9 Fig of the revised manuscript). Together, our newly introduced experimental data and the DepMap data support the proposition that the endogenous levels of A3A significantly dictate sensitivity to HMCES loss.

*2)	Both A3A and A3B have been implicated in cancer mutagenesis. This begs the question as to whether A3B overexpression also has a synthetic lethal interaction with HMCES knockout/down?   Addressing this may also help address point #1 above, as A3B is overexpressed in far more tumor cell lines than A3A.*

Recent data indicates that the effects of A3A and A3B are distinct in terms of their specificity and contributions to the mutagenic signatures(Chan et al. 2015; Mas Ponte and Supek 2020; Vohringer and Gerstung 2019). While we agree that this would be an interesting experiment, we feel that establishing the A3B inducible system in multiple cell lines is out of the scope of the current manuscript. We would also like to note that prior work from five different groups that used genomics (Chan et al. 2015; Mas Ponte and Supek 2020; Nik-Zainal et al. 2014) and experimental models (Cortez et al. 2019; Law et al. 2020) has provided abundant evidence that A3A, not A3B, is the more common mutagen in tumors of various cancer types including non-small cell lung cancers that we study herein.. We discuss this point to some extent in the revised discussion, particularly related to the finding that UNG2 was reported to be synthetically lethal in A3B expressing cells (Serebrenik et al. 2019), but that we found that UNG loss rescues the sensitivity of HMCES KO cells to A3A (Revised Fig 6B).

*3)	The authors cite a recent Cell Reports paper that was the first to demonstrate a synthetic lethal interaction between A3A and HMCES (ref 47). This paper should be a focal point in the discussion (similarities/differences, etc). I don't think this prior work ruins the overall impact of the present study as the extensive CRISPR screening data are valuable.*

We agree with this point and we have extended the discussion to compare/contrast this with our work and also include the data from our secondary screen (in an HMCES KO background) that helps to address the mechanism further. We also address the implications of some of the findings in our secondary screen with another recent paper that proposed HMCES loss was synthetic lethal with the loss of HR and TLS repair pathways(Srivastava et al. 2020), a finding that is not clearly replicated in our data (albeit in a different cell line). We hope that the revised discussion will allow the reader to better appreciate our data in the context of other recent work, as well as highlight some particularly interesting findings from our screens for future investigation.

*4)	Data accessibility - perhaps I missed it, but the supplement should include results for all genes covered in each CRISPR screen. The long term impact of this paper may be the large data sets that can be compared, contrasted, and followed-up by the broader community.*

We agree and as we mentioned in the reply to reviewer 1, we have provided annotated supplemental tables with both raw data (sgRNA read counts) and processed data (MAGECK log-fold change scores) for all experimental conditions to address this point (S2 Table, S6 Table and S9 Table in revised manuscript).

*Reviewer #3:*

*This paper describes the genetic connection between HMCES and the overexpression of APOBEC in lung cancer cells. Indeed, the authors identify HMCES as required to maintain viability in cancer cell with high levels of APOBEC. This synthetic lethality is observed directly in several lung cancer cell lines, but is validated further using available cancer expression data. Finally, the authors observed a synergistic effect of the combination of HMCES depletion with several DNA damaging agents and DNA repair factor inhibitors currently in use or in clinical trials for cancer treatment.*

*In general, the data presented in the paper are solid, the experiments are the appropriate, they are well executed and fully support the authors claim. Thus, scientifically I have no doubts of the manuscript validity. On the other hand, the main message of the paper is a compelling genetic interaction between APOBEC and HMCES. Although there is little insight on the molecular mechanisms behind this interaction, further than the known fact that HMCES protect abasic sites, it is clear that these observations might have relevance in cancer treatment. For this reviewer is hard to judge if the potential oncological treatment value is enough to overcome the lack of mechanistical information. Thus, what I can say is that scientifically I think the data support the main claims of the authors, but the editor should consider if the advance is enough to grant the publication in PLOS Biology*

We appreciate the positive comments from the reviewer about the work. To further address the mechanism, we have added new data from our secondary screen in HMCES KO cells (Reviewer Figure 1 and revised Fig 6). One of the hits that stands out is the *UNG* gene coding for the uracil-DNA glycosylases UNG1 and UNG2 (mitochondrial and nuclear, respectively). *UNG* loss promotes the viability of HMCES KO cells expressing A3A (i.e. it is positively selected) indicating that the production of AP-sites is critical for the toxicity of A3A in HMCES KO cells. We also identified the mismatch repair (MMR) linchpin protein MSH2 among the positively selected genes specific for HMCES KO expressing A3A. In recent work we proposed that the very common ‘mutation fog’ (‘omikli’) mutational pattern induced by A3A is MMR dependent and occurs in a manner associated with the replication forks(Mas Ponte and Supek 2020). The identification of MSH2 is consistent with this proposition and we have added this point to the discussion.

Among genes negatively selected specifically in the HMCES KO background, TDP1 was identified. TDP1 has been shown to process AP-sites for repair from yeast to humans and was proposed to play a primary role in resolving 3’ base lesions that can stall replication forks and lead to toxic DNA damage, particularly in BRCA1/2 deficient backgrounds (Lebedeva et al. 2012; Álvarez-Quilón et al. 2020). In addition, TDP1 is a widely explored target in oncology, given its primary role in processing Topoisomerase I-mediated damage, and is thus relevant to future clinical application of the work and the potential targeting of HMCES. As TDP1 did not come out in our initial screens (i.e. TDP1 is *not* A3A-conditionally essential in the HMCES wild-type background), this indicates that it provides backup for HMCES in preventing the toxicity of A3A-mediated damage. We have now added discussion about this and other selected hits from the secondary screen to the discussion as well, including VCPIP1, that is linked to the repair of multiple enzymes that repair DNA-protein crosslinks(Huang et al. 2020) and PPP2R2A, a candidate tumor suppressor that negatively regulates ATM-CHK2 signaling(Kalev et al. 2012; Ruvolo 2016).

*Other points:*

*1.	HMCES is, among the original candidates, the one that show a clear differential behavior regarding TP53 status. However, in the studies using available cancer data there is no mention to this protein status. Could the authors separate the data between TP53 positive and negatives and see if this dependence on this protein is validated?*

As we described in previous comments, we have addressed this point, analyzing the DepMap data and taking into account the p53 status (Reviewer Fig 3 and revised S9 Fig). One limitation is that few cell lines have wt TP53 compared to mutant. Increased HMCES dependency correlates quite clearly with A3A mutational signature in the cell line genome in the TP53 mutant, however this is not at all the case in the TP53 wild-type cells. (Reviewer Fig 3 and revised S9 Fig). We would also like to note that in response to Reviewer 1 point 3 we performed additional experiments to support that loss of TP53 sensitizes to HMCES inactivation in our isogenic A549 cell line pair (Reviewer Fig 4 and revised Fig 4F).

**Bibliography**

Álvarez-Quilón, A., Wojtaszek, J.L., Mathieu, M.-C., et al. 2020. Endogenous DNA 3’ blocks are vulnerabilities for BRCA1 and BRCA2 deficiency and are reversed by the APE2 nuclease. *Molecular Cell* 78(6), p. 1152–1165.e8.

Chan, K., Roberts, S.A., Klimczak, L.J., et al. 2015. An APOBEC3A hypermutation signature is distinguishable from the signature of background mutagenesis by APOBEC3B in human cancers. *Nature Genetics* 47(9), pp. 1067–1072.

Cortez, L.M., Brown, A.L., Dennis, M.A., et al. 2019. APOBEC3A is a prominent cytidine deaminase in breast cancer. *PLoS Genetics* 15(12), p. e1008545.

Hart, T., Brown, K.R., Sircoulomb, F., Rottapel, R. and Moffat, J. 2014. Measuring error rates in genomic perturbation screens: gold standards for human functional genomics. *Molecular Systems Biology* 10, p. 733.

Hart, T., Tong, A.H.Y., Chan, K., et al. 2017. Evaluation and Design of Genome-Wide CRISPR/SpCas9 Knockout Screens. *G3 (Bethesda, Md.)* 7(8), pp. 2719–2727.

Huang, J., Zhou, Q., Gao, M., et al. 2020. Tandem Deubiquitination and Acetylation of SPRTN Promotes DNA-Protein Crosslink Repair and Protects against Aging. *Molecular Cell* 79(5), p. 824–835.e5.

Jarvis, M.C., Ebrahimi, D., Temiz, N.A. and Harris, R.S. 2018. Mutation signatures including APOBEC in cancer cell lines. *JNCI Cancer Spectrum* 2(1).

Kalev, P., Simicek, M., Vazquez, I., et al. 2012. Loss of PPP2R2A inhibits homologous recombination DNA repair and predicts tumor sensitivity to PARP inhibition. *Cancer Research* 72(24), pp. 6414–6424.

Law, E.K., Levin-Klein, R., Jarvis, M.C., et al. 2020. APOBEC3A catalyzes mutation and drives carcinogenesis in vivo. *The Journal of Experimental Medicine* 217(12).

Lebedeva, N.A., Rechkunova, N.I., El-Khamisy, S.F. and Lavrik, O.I. 2012. Tyrosyl-DNA phosphodiesterase 1 initiates repair of apurinic/apyrimidinic sites. *Biochimie* 94(8), pp. 1749–1753.

Mas Ponte, D. and Supek, F. 2020. DNA mismatch repair promotes APOBEC3-mediated diffuse hypermutation in human cancers. *Nature Genetics*.

Mehta, K.P.M., Lovejoy, C.A., Zhao, R., Heintzman, D.R. and Cortez, D. 2020. HMCES Maintains Replication Fork Progression and Prevents Double-Strand Breaks in Response to APOBEC Deamination and Abasic Site Formation. *Cell reports* 31(9), p. 107705.

Mohni, K.N., Wessel, S.R., Zhao, R., et al. 2019. HMCES Maintains Genome Integrity by Shielding Abasic Sites in Single-Strand DNA. *Cell* 176(1–2), p. 144–153.e13.

Nik-Zainal, S., Wedge, D.C., Alexandrov, L.B., et al. 2014. Association of a germline copy number polymorphism of APOBEC3A and APOBEC3B with burden of putative APOBEC-dependent mutations in breast cancer. *Nature Genetics* 46(5), pp. 487–491.

Petljak, M., Alexandrov, L.B., Brammeld, J.S., et al. 2019. Characterizing mutational signatures in human cancer cell lines reveals episodic APOBEC mutagenesis. *Cell* 176(6), p. 1282–1294.e20.

Ruvolo, P.P. 2016. The broken “Off” switch in cancer signaling: PP2A as a regulator of tumorigenesis, drug resistance, and immune surveillance. *BBA clinical* 6, pp. 87–99.

Serebrenik, A.A., Starrett, G.J., Leenen, S., et al. 2019. The deaminase APOBEC3B triggers the death of cells lacking uracil DNA glycosylase. *Proceedings of the National Academy of Sciences of the United States of America* 116(44), pp. 22158–22163.

Shukla, V., Halabelian, L., Balagere, S., et al. 2020. HMCES Functions in the Alternative End-Joining Pathway of the DNA DSB Repair during Class Switch Recombination in B Cells. *Molecular Cell* 77(2), p. 384–394.e4.

Srivastava, M., Su, D., Zhang, H., et al. 2020. HMCES safeguards replication from oxidative stress and ensures error-free repair. *EMBO Reports* 21(6), p. e49123.

Vohringer, H. and Gerstung, M. 2019. Learning mutational signatures and their multidimensional genomic properties with TensorSignatures. *BioRxiv*.
