# Supplementary material for: Loss of the abasic site sensor HMCES is synthetic lethal with the activity of the APOBEC3A cytosine deaminase in cancer cells
Source: PLoS Biol. 2021 Mar 31;19(3):e3001176. doi: 10.1371/journal.pbio.3001176 (PMC8041192; doi:10.1371/journal.pbio.3001176)
Supplement: Attachment — Submitted filename: Reviewer rebuttal 2.docx [file pbio.3001176.s035.docx]

**Point-by-point responses (blue text) to reviewer comments (black text):**

Reviewer #1:

First I need to mention that I was not aware that the manuscript was under "scoop protection" during the initial submission, which explained some of my initial comments. That being said, the authors did a very good job addressing my comments and I am happy to recommend publication.

We would like to thank the reviewer again for their time and the comments that helped to strengthen the manuscript.

Reviewer #2:

In this work by Byana and colleagues performed a CRISPR screen to identify synthetic lethal combinations with high levels of APOBEC3A expression in cell lines derived from lung adenocarcinoma (A549 and LXF-289). They identified HMCES as permissive factor for A3A mutagenesis, since depletion of HMCES leads to A3A-driven cell death as a result of hypermutations and increased levels of DNA damage. Moreover, they demonstrate that this phenotype is dependent on the TP53 status of the cells. In fact, cells mutated or lacking functional p53 are more sensitive to the HMCES depletion to A3A mediated damage.

The majority of weak points of this manuscript were addressed in the first round of revisions and the authors answered to the majority of questions. Even though the synthetic lethality of A3A activity and HMCES depletion was previously described by the Cortez lab (Mehta et al, 2020, Cell Reports), the authors have notably improved the quality and novelty of the manuscript by performing a secondary CRISPR screen in HMCES KO cell lines. This screen provided insights into other genetic interactions of HMCES in the context of A3A activity and confirmed that the sensitivity of the depletion of HMCES is dependent on A3A activity. In fact, they show that A3A is among the top hits in this screen. Furthermore, they demonstrated that cancer cells are more sensitive to HMCES depletion when A3A expression is high and cells are lacking TP53.

Overall, the manuscript is well-revised and a strong contribution to the literature. My final (minor) point is that the discussion should be a bit better balanced to address why previously described A3A synthetic lethal combinations do not appear in the present work. For instance, multiple labs have reported a synthetic lethal interaction with A3A and ATR inhibition but, curiously, ATR does not appear to come up in any of the screens here (though it is used to help functionally validate the present work). These differences should be included in the discussion of the present manuscript.

We would like to thank the reviewer again for their careful reading of the manuscript and constructive comments that helped to improve the paper.

Regarding the discussion, we have made some revisions to address these points but will explain here in more detail what we think may explain the discrepancy between the inhibitor and screening data. Previous work, showed that ATRi and CHKi treatment sensitized A3A expressing cells[1,2], a result we also observed in Figure 5A with ATRi. However, it is true that ATR did not emerge as a hit in our primary screens (nor did ATM or PRKDC/DNA-PKcs, the inhibition of which also caused some sensitivity in LXF-289 cells; Fig 5). Here we provide the reviewer with a figure demonstrating the effects sgRNAs targeting ATR, CHK1, ATM and PRKDC in LXF-289 cells across conditions tested.

**Reviewer Fig 1: Summarized scores for DNA damage responsive kinases across LXF-289 samples.** The overall score for each kinase in the indicated dataset is plotted. Note that the samples in the gray box were not DOX treated to induce A3A (see text below for further explanation).

ATR and CHK1 are both common essential genes, a point that emerged originally from work in mice[3,4], and more recently in the DepMap data where ATR is essential in 794 of 808 cell lines and CHK1 (CHEK1) in 795 or 796 cell lines tested in the Avana21Q1 dataset (depmap.org). The fact that ATR and CHK1 are common essential genes likely makes it more difficult to detect them as conditionally essential genes in our screens due to the fact that they dropout the untreated cells to a similar extent early in the cell culture expansion. It is possible that if screens were performed on a shorter timescale, or with transient depletion, we would identify ATR and CHK1 as hits.

Recent data has highlighted that there is a mechanistic distinction between ATR/CHK1 loss and small molecule inhibition with regards to their relative severity in different contexts. It was demonstrated that a kinase dead allele of either ATR or CHK1 is mechanistically distinct from the KO, in that it leads to trapping of ATR and RPA retention on chromatin[5,6]. Assuming the inhibitors function in a similar way to the kinase dead alleles, this could lead to differential genetic dependencies on ATR/CHK1 loss vs inhibition. Similar work was performed for ATM where the kinase dead allele, in contrast to the full knockout, is essential[7,8]. Therefore, this could also explain why we see some effects of ATM and DNA-PKcs inhibitors but do not identify them as hits that differentially sensitized A3A expressing cells in the screens (Fig 5 and Reviewer Fig 1). We note that many PIKK regulators and targets were identified in the primary and secondary screens and we have annotated these in Fig 2E and Fig 6C of the revised manuscript.

Notably, in the LXF-289 HMCES KO cells, we did identify ATR and CHK1 as genes that reduced fitness when depleted (Reviewer Fig 1). However, note that, like in the parental lines, there is little difference whether A3A is induced or not (compare to non-DOX treated in gray box). In other words, the difference is a result of HMCES status. As we showed in the damage sensitivity data, the HMCES KO line is already less fit than the parental line (Fig 5) and it may be sensitized to ATR/CHK1 loss compared to the parental line (ex. more rapid death and depletion from the pool), but A3A expression is not severe enough to detectably modify the phenotype in this experiment. Alternatively, the effect of A3A may be due to specific effects of the inhibitors that are not reproduced by deletion of the gene. For example, if ATR/CHK1 inhibition stabilizes ssDNA, that could provide additional substrate for A3A modification, increasing the extent of damage[5].

Some of the data from HMCES KO lines not expressing A3A was referred to in the previous discussion but not provided in the previous submission, as we focused on the effects of A3A. We have now included this comparison in a summarized form to support the discussion points (S12 Fig) that compare our results with that of the Chen lab and the tables in full for further analysis (S12 Data)[9].

We think that the Reviewer raises an interesting point and for the purposes of addressing this in the text, we have made the following modifications below that tackle this in less detail than we have here, but we believe address the main points necessary for the discussion. In addition, we exchanged the secondary screen plot that was in previous S11C Fig for Fig 6C, to allow readers to more readily identify the genes in the secondary screen that give advantage or lethality specifically in HMCES KO cells expressing A3A. We also annotated this, as well as the Fig 2E (summarized primary screen hits) to show targets or regulators of the PIKKs that are potentially relevant to the PIKK inhibitor sensitivities that we and others have observed.

We appreciate the reviewer pointing out this issue, as I think it will be important to address further going forward, and we hope this will sufficiently address the concern for the purposes of the manuscript.

**Modified discussion related to the reviewer comments:**

Inhibition of the ATR-CHK1 kinases, that activate cell cycle checkpoints in S and G2 in response to replication stress, was shown to enhance A3A-mediated toxicity in AML, lung, ovarian and osteosarcoma cell lines[32,33]. We did not identify either kinase in our primary screens, however both genes are common essential genes in a large panel of cancer cell lines tested (DepMap.org), likely making them more difficult to detect as conditionally essential genes. Acute inhibition of ATR or CHK1 is better tolerated than genetic deletion or kinase dead alleles, that result in embryonic lethality, suggesting it may a more sensitive approach[82–85]. We did identify ATR as a hit in our LXF-289 HMCES KO cells that were not treated with DOX (no A3A) compared to parental cells (S12 Fig). This would potentially remove these cells from the pool early due to the HMCES KO interaction, thus precluding the identification of ATR following A3A treatment in those cells. Kinase dead alleles of both ATR and CHK1 have also been demonstrated to elicit phenotypes distinct from genetic deletion[82–85]. This suggests the possibility that the interaction between A3A and ATR/CHK1 inhibitors could be due to specific effects of the inhibitors, such as increased ssDNA stability or exposure. Regardless, our results further extend the robustness of previous observations to additional lung cancer cell lines, as we observed that A3A expression provoked particularly strong sensitivity to ATR inhibitors, among the interventions tested (Fig 5A)[32,33].

In contrast to previous work, we also found increased sensitivity to ATM and DNA-PKcs inhibitors in LXF-289 NSCLCs lacking HMCES, again likely reflecting differences in the genetic backgrounds analyzed[78]. While neither kinase is generally essential like ATR, and neither was identified as a strong hit in the screening, multiple PIKK substrates and regulators were identified, supporting the potential roles of these signaling pathways in survival (Figs 2E and 6C). As inhibitors for the PIKKs are in multiple clinical trials, targeting HMCES may represent a strategy to enhance their efficacy, particularly in combination with radiotherapy. This may be particularly potent in cancers with *PPP2R2A* deletions[77]. PPP2R2A is a negative regulator of ATM-CHK2 and a candidate tumor suppressor gene commonly deleted in ovarian, prostate, liver and bladder cancers[76,77,86]. Loss of PPP2R2A enhanced toxicity of A3A in HMCES KO cells (Figs 6B and 6C), and depletion or PPP2R2A was shown to enhance the toxicity of ATR-CHK1 or PARP inhibitors in NSCLC[86,87].

Of the secondary screen hits that were specific to HMCES KO cells expressing A3A, TDP1 stood out as the primary negatively selected DNA repair protein (Fig 6B and 6C). TDP1 plays a key role in resolving blocked 3’-ends, including TOP1 (Topoisomerase I) DPCs[73]. The processing of TOP1-DPCs by TDP1 is preceded by the proteolytic degradation of TOP1 by the SPRTN and VCP/p97 proteases(REF). Following its activation by ATM/ATR, VCPIP1, another strong hit in the secondary screen, deubiquitinates the SPRTN metalloprotease to promote its activation and the removal of DNA-protein crosslinks (DPCs) that can cause damage during DNA replication[75,88]. TDP1 can then resolve the digested 3’-TOP1-DPC to facilitate DNA repair. TDP1 has also been shown to act as an AP-lyase and is essential in cells lacking APE1, which promotes the repair of AP-sites by BER, indicating it may play a wider role in the repair of A3A-mediated damage[74,89,90]. TDP1 inhibitors are currently being explored in clinical trials to sensitize cells to topoisomerase inhibitors and could be also used in conjunction with HMCES depletion/inhibition to sensitize cancer cells to APOBEC3-expression and perhaps other agents that generate AP-sites[91].

**Bibliography**

Brown, E.J. and Baltimore, D. 2000. ATR disruption leads to chromosomal fragmentation and early embryonic lethality. *Genes & Development* 14(4), pp. 397–402.

Buisson, R., Lawrence, M.S., Benes, C.H. and Zou, L. 2017. APOBEC3A and APOBEC3B activities render cancer cells susceptible to ATR inhibition. *Cancer Research* 77(17), pp. 4567–4578.

Daniel, J.A., Pellegrini, M., Lee, B.-S., et al. 2012. Loss of ATM kinase activity leads to embryonic lethality in mice. *The Journal of Cell Biology* 198(3), pp. 295–304.

Green, A.M., Budagyan, K., Hayer, K.E., et al. 2017. Cytosine deaminase APOBEC3A sensitizes leukemia cells to inhibition of the DNA replication checkpoint. *Cancer Research* 77(17), pp. 4579–4588.

Kawale, A.S. and Povirk, L.F. 2018. Tyrosyl-DNA phosphodiesterases: rescuing the genome from the risks of relaxation. *Nucleic Acids Research* 46(2), pp. 520–537.

Liu, Q., Guntuku, S., Cui, X.S., et al. 2000. Chk1 is an essential kinase that is regulated by Atr and required for the G(2)/M DNA damage checkpoint. *Genes & Development* 14(12), pp. 1448–1459.

Menolfi, D., Jiang, W., Lee, B.J., et al. 2018. Kinase-dead ATR differs from ATR loss by limiting the dynamic exchange of ATR and RPA. *Nature Communications* 9(1), p. 5351.

Muralidharan, S.V., Nilsson, L.M., Lindberg, M.F. and Nilsson, J.A. 2020. Small molecule inhibitors and a kinase-dead expressing mouse model demonstrate that the kinase activity of Chk1 is essential for mouse embryos and cancer cells. *Life Science Alliance* 3(8).

Qiu, Z., Fa, P., Liu, T., et al. 2020. A Genome-Wide Pooled shRNA Screen Identifies PPP2R2A as a Predictive Biomarker for the Response to ATR and CHK1 Inhibitors. *Cancer Research* 80(16), pp. 3305–3318.

Ruvolo, P.P. 2016. The broken “Off” switch in cancer signaling: PP2A as a regulator of tumorigenesis, drug resistance, and immune surveillance. *BBA clinical* 6, pp. 87–99.

Serebrenik, A.A., Starrett, G.J., Leenen, S., et al. 2019. The deaminase APOBEC3B triggers the death of cells lacking uracil DNA glycosylase. *Proceedings of the National Academy of Sciences of the United States of America* 116(44), pp. 22158–22163.

Srivastava, M., Su, D., Zhang, H., et al. 2020. HMCES safeguards replication from oxidative stress and ensures error-free repair. *EMBO Reports* 21(6), p. e49123.

Yamamoto, K., Wang, Y., Jiang, W., et al. 2012. Kinase-dead ATM protein causes genomic instability and early embryonic lethality in mice. *The Journal of Cell Biology* 198(3), pp. 305–313.
